# Cardiorespiratory fitness, muscular strength and risk of type 2 diabetes: a systematic review and meta-analysis

**DOI:** 10.1007/s00125-019-4867-4

**Published:** 2019-04-23

**Authors:** Jakob Tarp, Andreas P. Støle, Kim Blond, Anders Grøntved

**Affiliations:** 1 Research Unit for Exercise Epidemiology, Centre of Research in Childhood Health, Department of Sports Science and Clinical Biomechanics, University of Southern Denmark, Odense, Denmark; 2 Department of Sports Medicine, Norwegian School of Sports Sciences, Sognsveien 220, 0806 Oslo, Norway; 3 Department of Sports Science and Clinical Biomechanics, University of Southern Denmark, Odense, Denmark; 4 Center for Clinical Research and Prevention, Bispebjerg and Frederiksberg Hospital, The Capital Region, Frederiksberg, Denmark

**Keywords:** Epidemiology, Fitness, Meta-analysis, Physical activity, Public health, Systematic review, Type 2 diabetes

## Abstract

**Aims/hypothesis:**

The study aimed to quantitatively summarise the dose–response relationships between cardiorespiratory fitness and muscular strength on the one hand and risk of type 2 diabetes on the other and estimate the hypothetical benefits associated with population-wide changes in the distribution of fitness.

**Methods:**

We performed a systematic review with meta-analysis. The PubMed and EMBASE electronic databases were searched from inception dates to 12 December 2018 for cohort studies examining the association of cardiorespiratory fitness or muscular strength with risk of incident type 2 diabetes in adults. The quality of included studies was evaluated using the Newcastle–Ottawa Scale.

**Results:**

Twenty-two studies of cardiorespiratory fitness and 13 studies of muscular strength were included in the systematic review with both exposures having ten estimates available for the primary adiposity- or body size-controlled meta-analysis. In random-effects meta-analysis including 40,286 incident cases of type 2 diabetes in 1,601,490 participants, each 1 metabolic equivalent (MET) higher cardiorespiratory fitness was associated with an 8% (95% CI 6%, 10%) lower RR of type 2 diabetes. The association was linear throughout the examined spectrum of cardiorespiratory fitness. In 39,233 cases and 1,713,468 participants each 1 SD higher muscular strength was associated with a 13% (95% CI 6%, 19%) lower RR of type 2 diabetes. We estimated that 4% to 21% of new annual cases of type 2 diabetes among 45–64-year-olds could be prevented by feasible and plausible population cardiorespiratory fitness changes.

**Conclusions/interpretation:**

Relatively small increments in cardiorespiratory fitness and muscle strength were associated with clinically meaningful reductions in type 2 diabetes risk with indication of a linear dose–response relationship for cardiorespiratory fitness.

**Registration:**

PROSPERO (CRD42017064526).

**Electronic supplementary material:**

The online version of this article (10.1007/s00125-019-4867-4) contains peer-reviewed but unedited supplementary material, which is available to authorised users.

## Introduction

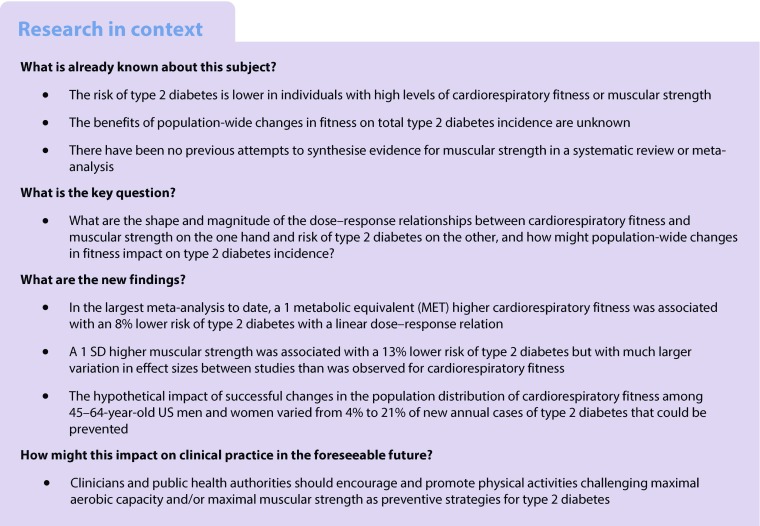



Type 2 diabetes prevention is a key health priority of the 21st century [1, 2]. Lifestyle interventions including engagement in physical activity are established primary [3, 4] and secondary preventive strategies [5]. These lifestyle interventions are supported by evidence from observational studies convincingly showing that engagement in higher levels of physical activity is associated with lower risk of subsequent type 2 diabetes [6, 7]. Physical activity influences both cardiorespiratory fitness [8], which reflects the capacity of the cardiovascular system, and muscular fitness [9], which is a construct encompassing muscular strength, power and endurance [10]. Cardiorespiratory and muscular fitness phenotypes can be accurately and precisely quantified [10, 11] using methods that are feasible in a clinical setting [12]. Determining the dose–response relationships of these objective markers with incidence of type 2 diabetes may help provide tangible targets for individual and population physical activity prescriptions and interventions.

Attempts to quantitatively summarise the association between cardiovascular fitness and risk of type 2 diabetes from prospective cohort studies [6, 13] have been limited by (1) assuming a linear dose–response association [13]; (2) pooling estimates with and without control for adiposity [13] despite substantial attenuation of fitness–diabetes associations when adiposity is controlled for [14–16]; and (3) not exploring other sources of heterogeneity [6, 13]. A recent meta-analysis addressed these limitations [17] but did not translate their findings into absolute public health metrics. Such metrics are needed to guide allocation of public health resources. Further, a landmark study including more than one million individuals [18] was not included in the meta-analysis. No previous systematic review or meta-analysis of the association between muscular fitness and type 2 diabetes incidence exists.

The purpose of this study was to (1) systematically review and meta-analyse prospective cohort studies reporting on the association of cardiovascular and muscular fitness with the risk of incident type 2 diabetes; (2) investigate sources of heterogeneity including the importance of adjustment for adiposity; and (3) inform public health policy by estimating relevant absolute risk metrics of the population-wide impact of fitness on type 2 diabetes risk.

## Methods

The study protocol was registered with PROSPERO (CRD42017064526) and reported according to Meta-analysis Of Observational Studies in Epidemiology (MOOSE) guidelines. Ethics approval was not required.

### Data sources and searches

PubMed and EMBASE were searched for cohort studies on the associations among cardiorespiratory fitness, muscular strength and risk of incident type 2 diabetes (electronic supplementary material [ESM] Table 1 and ESM Table 2). No restrictions on date of publication were set. The final search was conducted on 12 December 2018. We additionally went through reference lists from studies included in the review and searched Web of Science for studies citing these publications.

### Study selection

Cohort studies were eligible if they (1) followed individuals free of type 2 diabetes at baseline, but we included cohorts of individuals with diabetes-associated conditions (e.g. dyslipidaemia or obesity); (2) assessed cardiorespiratory fitness using a maximal or sub-maximal test of any form at baseline or assessed muscular strength using a test requiring a maximal effort at baseline; (3) considered incidence of type 2 diabetes as an isolated outcome; and (4) were published in Scandinavian or English language. Conference abstracts were included if relevant. Restriction to muscular fitness operationalised by maximal strength (thereby excluding muscular power and endurance) [10] was chosen to maximise the potential for exposure harmonisation. Studies were excluded if they (1) considered a cohort of individuals with a chronic disease (e.g. cancer); or (2) assessed muscular endurance or power (full details in ESM Table 3). To be included in meta-analysis, studies additionally had to provide (1) HR, OR or RR for type 2 diabetes for one or more cardiorespiratory fitness or muscular strength estimates (linear or categorical); (2) estimates of variance or data to calculate it; and (3) had cardiorespiratory fitness estimates convertible to metabolic equivalents (METs) [10] or muscular strength estimates convertible to per SD. Two researchers (J. Tarp and A. P. Støle) independently screened titles and abstracts using Endnote X7.7.1 (Clarivate Analytics, PA, USA) according to pre-specified criteria. When eligibility was ambiguous, the full text was retrieved. Disagreement was resolved by discussion including a third researcher (A. Grøntved).

### Data extraction and quality assessment

Data from eligible studies were independently extracted by two researchers (J. Tarp and A. P. Støle) using a piloted template. Disagreements were resolved by discussion. The following were extracted if available: first author, country, cohort name/title, cohort recruitment period (years), sex, ethnicity, baseline age, length of follow-up, method of outcome ascertainment, cumulative diabetes incidence, method of exposure ascertainment, levels of exposure, case count and total participant count in fitness categories, HRs/ORs/RRs and associated variance for linear or categorical estimates, and control variables applied in retrieved estimates. From each study or cohort we extracted estimates with and without control for an index of adiposity if available. We extracted adiposity- and non-adiposity-controlled estimates from the same report if possible, but included other reports from the same cohort if this increased sample sizes or the number of cases or facilitated a more direct BMI contrast. When a study did not provide either (1) estimates with and without adiposity control; or (2) estimates from at least two categories compared with a common reference, we contacted corresponding authors and requested additional information using a standardised template (template available on request to the corresponding author). The Newcastle–Ottawa Scale (NOS) [19] with modifications informed by the study question was used to rate overall study quality (details of the NOS rating and criteria in ESM Table 4 and ESM Table 5). Age, sex, ethnicity, cardiorespiratory/muscular fitness, smoking [20], family history of diabetes, dietary intake [21], alcohol consumption [22], TV viewing [23] and socioeconomic status were considered putative confounding variables which could potentially result in biased measures of association. When multiple publications from the same cohort were identified, we used the manuscript presenting the largest case and participant count with harmonisable exposure data (table of overlapping cohorts presented in ESM Table 6). If additional data (e.g. linear/categorical or with/without control for adiposity) from the cohort were available in other publications, we retrieved estimates from both of these papers.

### Data synthesis

A detailed overview of assumptions, calculations and unpublished data provided by contacted authors used in exposure harmonisation is provided in ESM Table 7 and ESM Table 8. If possible, cardiorespiratory fitness estimates were converted to METs for the non-linear analysis and to per 1 MET increase for the linear meta-analysis. Muscular strength was converted to per SD increase. Harmonisation of linear estimates was performed using transformation of the log-ratio estimate (using the natural logarithm) [24] under the assumption of fitness measures following a normal distribution and a log-linear association with type 2 diabetes incidence. Estimates for cardiorespiratory fitness based on treadmill duration were converted using exercise protocol-appropriate equations [25]. When the exposure level was unclear, but distributional assumptions allowed estimation, we assumed an SD of cardiorespiratory fitness of 2.0 METs [26, 27] in subsequent calculations. Cardiorespiratory fitness presented as watts per kg was converted to ml O_2_ kg^−1^ min^−1^ using a linear equation based on measurement of maximal oxygen uptake by indirect calorimetry (variance explained was 71% [ESM Table 7]). Data provided as ml O_2_ kg^−1^ min^−1^ were converted to METs by dividing by 3.5 [10]. When original estimates did not have the lowest fitness category as reference, we converted the lowest fitness level to the reference using the Hamling method [28].

### Statistical analysis

Harmonised estimates for cardiorespiratory and muscle strength were pooled using a random-effects model [29] under the assumption of a linear dose–response relationship and that each study provides an estimate in a distribution of ‘true’ estimates. We provide fixed-effects estimates for comparison. If studies provided HRs/RRs/ORs pertinent to a continuous form we used this estimate. Otherwise, and if at least two categories with a common reference were available, we used generalised least squares trend (GLST) estimation to estimate the study-specific dose–response association [30] taking into account the common reference group [31] and the non-zero exposure in the reference group by centring the exposure corresponding to the natural log RR [32]. In addition, we modelled the dose–response association between cardiorespiratory fitness and type 2 diabetes using restricted cubic splines with knots placed at the 25th, 50th and 75th percentiles [33] of the cardiorespiratory fitness distribution in the data. Departure from linearity was assessed by a Wald test examining the null hypothesis that the coefficient of the second spline was equal to zero [34]. At least two categories with a common reference were needed to be included in non-linear analysis. Insufficient data precluded non-linear analysis of muscular strength. Analyses were performed separately on estimates reported with and without inclusion of adiposity indices as control variables. Adiposity-adjusted estimates were included as primary analysis as we consider this the more conservative analysis. We present data for men and women separately if available but used a fixed-effects meta-analysis to pool estimates within a study based on other stratifications if relevant. ORs were assumed to approximate RRs [35]. Between-study heterogeneity was formally assessed using *I*^2^ as a measure of the proportion of variance not explained by random error and by visual interpretation of the forest plots. Sources of heterogeneity were explored by stratification on cohort and participant characteristics. Robustness of estimates was assessed by repeating the analysis excluding a single study at a time. Risk of small-study bias was investigated by funnel plot and Egger’s test for asymmetry. Estimates are presented with a 95% CI. Assuming that estimates represent causal effects, we calculated the risk difference for a 1 MET and a 1 SD increase in cardiorespiratory fitness and muscular strength, respectively. The risk differences (with 95% CIs) were calculated using the formula: risk difference = background incidence rate × (RR − 1) [36]. We calculated risk differences based on background annual rates in the age strata 18–44, 45–65 and ≥ 65 years based on 2015 US incidence data [37]. We calculated potential impact fractions (PIFs) [38, 39] as a measure of the percentage of new annual diabetes cases in the population that could hypothetically be prevented by interventions affecting the population distribution of cardiorespiratory fitness. Our hypothetical interventions were modelled on sex-specific population estimates of cardiorespiratory fitness for 40–59-year-olds from the US Fitness Registry and the Importance of Exercise National Database (FRIEND) [40]. PIFs were calculated under four counterfactual scenarios: (1) a structural intervention resulting in a 1 MET increase in the bottom 50% of the cardiorespiratory fitness distribution; (2) the same intervention but resulting in a population-wide 1 MET fitness increase; (3) achievement of the same cardiorespiratory fitness distribution as observed in age-matched individuals from the Norwegian population-based HUNT study [41]; and (4) achievement of cardiorespiratory fitness distribution identical to the most physically active tertile of age-matched individuals from the HUNT study [41]. Additional details are given in ESM Table 9. We were unable to calculate PIFs for muscular strength as no reference distribution was identified. All *p* values were two-sided and interpreted at the 0.05 level. Analyses were performed in Stata 15.0 (StataCorp, College Station, TX, USA).

## Results

### Literature search

**Fig. 1:**
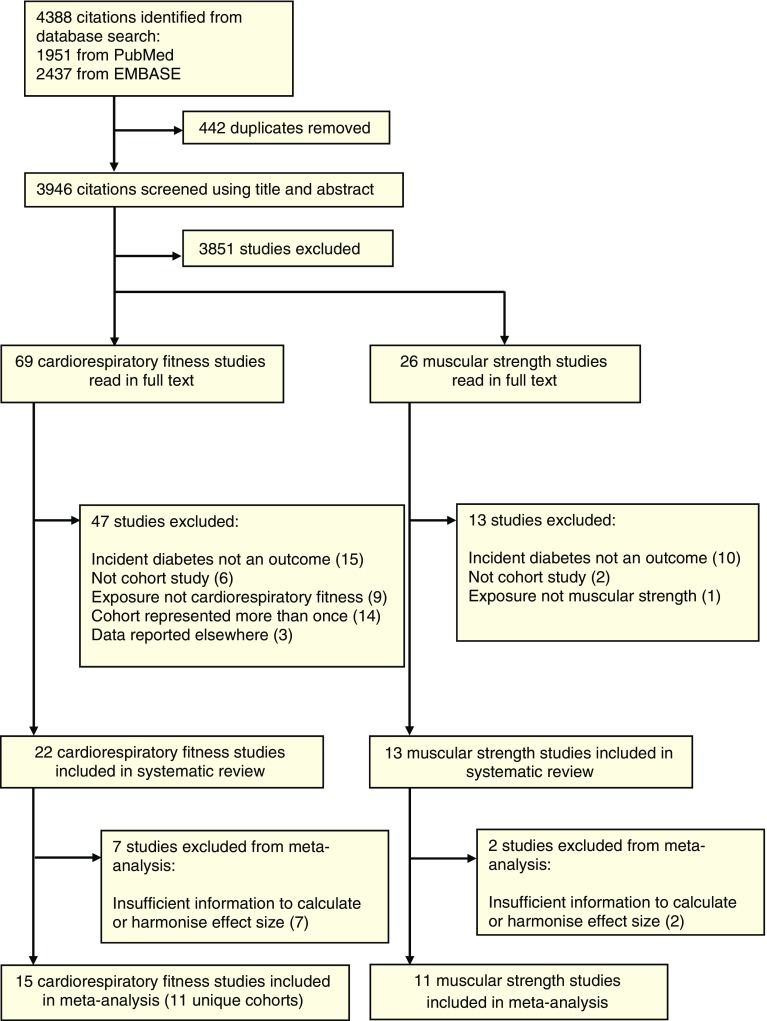
Flowchart of retrieved publications

In total, 22 studies (representing 18 unique cohorts) on cardiorespiratory fitness [13, 14, 18, 42–60] and 13 studies on muscular strength [15, 16, 18, 43, 61–69] were identified for inclusion in the systematic review. The phases of the literature search are shown in Fig. 1. Additional data were retrieved by personal communication to six cohorts for cardiorespiratory fitness [18, 45, 48–50, 52] and five for muscular strength [16, 18, 63, 65, 68].

### Study characteristics

Cohort studies reporting estimates for cardiorespiratory fitness ranged in size from 571 to 1,534,425 participants with a median cohort baseline age of 45 years. The cumulative incidence of type 2 diabetes ranged from 0.7% to 26.2% with median follow-up ranging from 3 to 29 years. Nine cohorts used a maximal test while eight used a sub-maximal assessment of fitness. For muscular strength, cohort sizes ranged from 328 to 1,534,425 participants. The median cohort age at baseline was 52 years. The cumulative incidence of type 2 diabetes ranged from 2.1% to 18.5% with median duration of follow-up ranging from 3 to 26 years. Eight studies reported muscle strength normalised to kilogram body weight (two of these after receiving additional data from study authors) while five did not normalise muscular strength. Eleven studies used maximal handgrip strength to define muscular strength while two used a composite index including multiple muscle groups. The median SD of muscular strength as a percentage of the mean was 31% (range: 22–39%). Of the 31 studies considered, only four used a measure other than BMI to control for adiposity. The median NOS score was six for cardiorespiratory fitness and six for muscular strength. Importantly, all studies failed to account for at least four of the seven pre-specified confounding factors with potentially non-trivial impact on the internal validity of risk estimates. An overview of study characteristics is presented in ESM Table 10 and ESM Table 11. Seven studies for cardiorespiratory fitness [54–60] and two studies for muscular strength [67, 69] presented data that were not harmonisable for inclusion in meta-analysis. Results from these studies generally supported a protective effect of higher cardiorespiratory fitness and muscular strength on risk of type 2 diabetes.

### Association between cardiorespiratory fitness and risk of type 2 diabetes

**Fig. 2:**
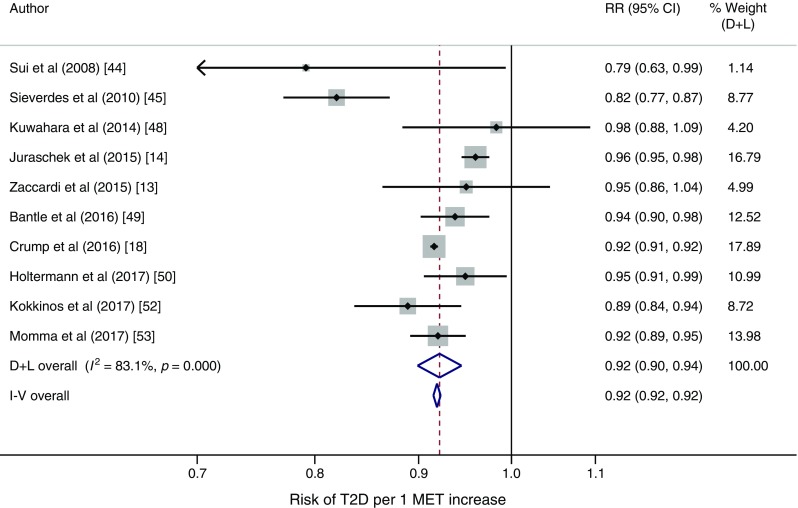
Study-specific RRs per 1 MET increase in cardiorespiratory fitness in models controlling for adiposity. Study weights are from the random-effects analysis (D+L). Pooled RRs from the random-effects analysis (D+L) and the fixed-effects analysis (I-V) are shown based on ten cohorts providing adiposity-controlled estimates. Four of these cohorts provided estimates per 1 MET (or ml O_2_ kg^−1^ min^−1^, converted to METs) [13, 14, 18, 50] while the linear estimate was modelled using GLST in six studies [44, 45, 48, 49, 52, 53]. D+L, DerSimonian and Laird (random-effects model); I-V, inverse variance (fixed-effects model)

In adiposity-controlled models including 40,286 incident cases of type 2 diabetes in 1,601,490 participants, each 1 MET higher cardiorespiratory fitness was associated with an 8% (95% CI 6%, 10%) reduction in risk of type 2 diabetes [13, 14, 18, 44, 45, 48–50, 52, 53]. The per 1 MET risk reduction in models omitting adiposity control was 20% (95% CI 14%, 25%) [14, 43–48, 50–52]. Study-specific per 1 MET estimates are shown as forests plots with (Fig. 2) and without (ESM Fig. 1) adiposity control.

**Fig. 3:**
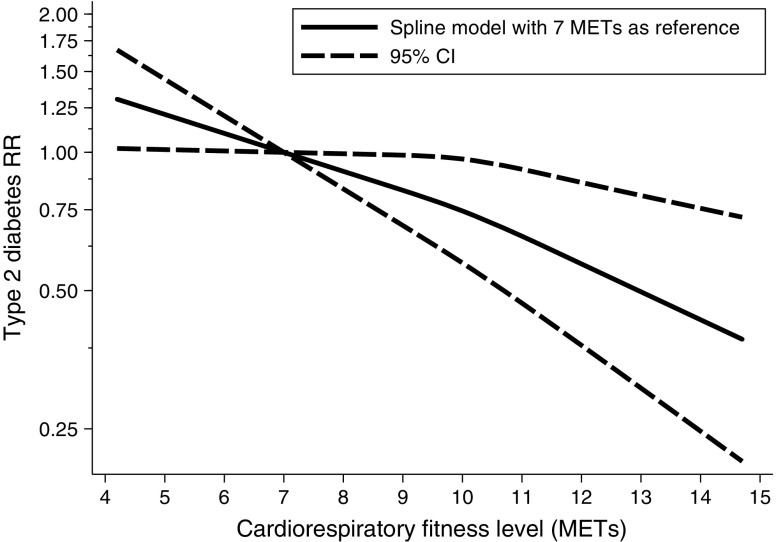
RR of type 2 diabetes with increasing cardiorespiratory fitness level modelled using restricted cubic splines. Estimates controlled for adiposity. The *y*-axis is natural log-transformed to maintain symmetrical CIs

The non-adiposity-controlled meta-analysis was highly sensitive to the choice of random- or fixed-effects modelling; estimated heterogeneity was substantial in both models (*I*^2^ 83% and 93%, respectively), whereas visual inspection of the forest plots suggested moderate heterogeneity. Heterogeneity appeared to be explained by the influence of two studies, one with a noticeably narrow 95% CI [18] and one with a substantial effect size and a moderate size CI [45]. Excluding these studies reduced the *I*^2^ to 48%. The association between adiposity-controlled cardiorespiratory fitness and type 2 diabetes risk modelled using restricted cubic splines [14, 18, 42, 44, 45, 48–50, 52, 53] is shown in Fig. 3. The model was consistent with a continued linear risk reduction with no statistical support of a non-linear association (*p* = 0.07) within our data ranging from four to 15 METs. A restricted cubic spline model including non-adiposity-controlled estimates [14, 44, 45, 48, 50–52] is presented in ESM Fig. 2.

The risk differences associated with a 1 MET higher cardiorespiratory fitness were 87 (95% CI 64, 112) incident diabetes cases per 100,000 people per year in the US population aged 45–64 years for the adiposity-controlled RR and 218 (95% CI 150, 280) for the non-adiposity-controlled RR (risk difference for other age strata available in ESM Table 12). The PIFs (adiposity-controlled) associated with a hypothetical intervention resulting in a 1 MET increase among the least fit 50% of US men and women aged 40–59 years were 4.2% and 3.6%, respectively. PIFs (adiposity-controlled) of achieving the same cardiorespiratory fitness distribution as is observed in a population-based sample of same-aged Norwegians were 18.0% and 20.6% for men and women, respectively (Table 1 [70, 71]); PIFs based on the non-adiposity-controlled estimate are presented in ESM Table 9. The impact of stratification on the pooled RR (adiposity-controlled) is shown in Table 2. Apart from a marked difference in sex-stratified results (only one study included women only), none of our stratification parameters substantially altered the risk reduction.

**Table 1:** PIFs and PAFs for counterfactual cardiorespiratory fitness distributions in 40–59-year-old US men and women

Intervention	Sex	Observed CRF distribution [40]	RR per 1 MET	Counterfactual CRF distribution	PIF
1 MET CRF increase achieved in the least fit 50%	Men	FRIEND database (US)^a^ Mean 10.37 SD 2.76	0.92 (adiposity-controlled)	Mean 10.82 SD 2.38	4.2%
1 MET CRF increase achieved in the least fit 50%	Women	FRIEND database (US)^a^ Mean 7.45 SD 2.05	0.92 (adiposity-controlled)	Mean 7.86 SD 1.68	3.6%
1 MET CRF increase achieved irrespective of initial CRF	Men	FRIEND database (US)^a^ Mean 10.37 SD 2.76	0.92 (adiposity-controlled)	Mean 11.37 SD 2.76	7.9%
1 MET CRF increase achieved irrespective of initial CRF	Women	FRIEND database (US)^a^ Mean 7.45 SD 2.05	0.92 (adiposity-controlled)	Mean 8.45 SD 2.05	7.8%
Achieve same CRF distribution as age-matched Norwegian population-based sample^b^	Men	FRIEND database (US)^a^ Mean 10.37 SD 2.76	0.92 (adiposity-controlled)	Norwegian HUNT study [41] (men aged 40–59 years) Mean 12.69 SD 2.31	18.0%
Achieve same CRF distribution as age-matched Norwegian population-based sample^b^	Women	FRIEND database (US)^a^ Mean 7.45 SD 2.05	0.92 (adiposity-controlled)	Norwegian HUNT study [41] (women aged 40–59 years) Mean 10.24 SD 1.92	20.6%
Achieve same CRF distribution as most active tertile of age-matched individuals from a Norwegian population-based sample^c^	Men	FRIEND database (US)^a^ Mean 10.37 SD 2.76	0.92 (adiposity-controlled)	Norwegian HUNT study [41] (men aged 40–59 years) Mean 14.09 SD 2.31	26.8%
Achieve same CRF distribution as most active tertile of age-matched individuals from a Norwegian population-based sample^c^	Women	FRIEND database (US)^a^ Mean 7.45 SD 2.05	0.92 (adiposity-controlled)	Norwegian HUNT study [41] (women aged 40–59 years) Mean 11.19 SD 2.08	26.2%
Elimination of ‘unfit’ category (bottom 25% of CRF)	Men	FRIEND database (US)^a^ Mean 10.45 SD 2.77	0.92 (adiposity-controlled)	–	PAF^d^ 8.1%
Elimination of ‘unfit’ category (bottom 25% of CRF)	Women	FRIEND database (US)^a^ Mean 7.45 SD 2.05	0.92 (adiposity-controlled)	–	PAF^d^ 5.9%

^a^Age groups combined using the Cochrane Handbook for Systematic Reviews of Interventions, Table 7.7.a: Formulae for combining groups [70]

^b^‘Feasible minimum risk’

^c^‘Plausible minimum risk’

^d^PAFs [71] for low cardiorespiratory fitness were calculated by defining the bottom 25% of the population CRF distribution as unfit (<8.4 METs would be classified as unfit for men whereas women with a CRF <6.0 METs would be classified as unfit) based on the US FRIEND database at 40–59 years of age. We then estimated the proportion of total diabetes cases that could theoretically be prevented by changing the cardiorespiratory fitness level of all unfit adults to the fitness level matching the distribution of the population of ‘fit’ individuals (≥25th percentile). RRs were based on a contrast between the fitness level of the sex-specific 12.5th percentile (the midpoint of the 1st to 25th percentile interval) and the 62.5th percentile (the midpoint of the 25th to 99th percentile) estimated from the restricted cubic spline model. This analysis is comparable to conventional PAF calculations based on eliminating the exposure and ‘shifting’ exposed individuals into matching the distribution of the ‘non-exposed’ reference category (above the sex-specific MET cut-points as specified above)

CRF, cardiorespiratory fitness; PAF, population attributable fraction

**Table 2:** RR of type 2 diabetes stratified by cohort and population characteristics

Variable	Estimates included	RR per 1 MET	RR per SD	95% CI	I^2^ (%)	Incident type 2 diabetes cases
Cardiorespiratory fitness						
Exposure assessment						
Sub-maximal	3	0.93		0.91, 0.96	8	2212
Maximal	7	0.91		0.88, 0.94	88	38,074
Work performed on						
Treadmill	5	0.90		0.85, 0.95	87	3913
Bicycle ergometer	5	0.92		0.91, 0.93	10	36,373
Outcome assessment						
Clinical assessment	5	0.93		0.91, 0.95	0	2383
Registry	4	0.93		0.90, 0.96	91	37,314
Self-report	1	0.95		0.77, 0.87	-	589
Region						
North America	5	0.90		0.85, 0.95	87	3913
Scandinavia	3	0.92		0.90, 0.94	26	34,679
Japan	2	0.93		0.89, 0.98	27	1694
Sex						
Men only	7	0.91		0.89, 0.94	67	38,037
Women only	1	0.79		0.63, 0.99	-	143
NOS						
7–8 stars awarded	4	0.92		0.91, 0.92	3	35,379
≤6 stars awarded	6	0.93		0.89, 0.96	83	4907
Muscular strength						
Normalisation of muscular strength						
No normalisation	5		0.95	0.87, 1.04	65	3598
Per kg body weight	7		0.83	0.79, 0.86	68	35,635
Assessment type						
Maximal handgrip strength	10		0.86	0.79, 0.94	79	4996
Other methods	2		0.93	0.72, 1.21	92	34,237
Outcome assessment						
Clinical assessment	9		0.88	0.79, 0.98	83	2070
Registry	2		0.87	0.78, 0.97	79	36,947
Self-report	1		0.78	0.65, 0.95	-	216
Region						
United States	5		0.98	0.88, 1.08	65	724
Other	7		0.82	0.77, 0.87	47	38,509
Sex						
Men only	4		0.81	0.77, 0.88	0	34,986
Women only	3		0.88	0.72, 1.09	83	468
Age group						
<60 years	8		0.87	0.80, 0.95	85	38,431
≥60 years	4		0.88	0.73, 1.06	72	802
NOS						
7–8 stars awarded	5		0.84	0.76, 0.94	88	35,099
≤6 stars awarded	7		0.90	0.80, 1.01	70	4134

Estimates are from random-effects models controlling for adiposity

### Association between muscular strength and risk of type 2 diabetes

**Fig. 4:**
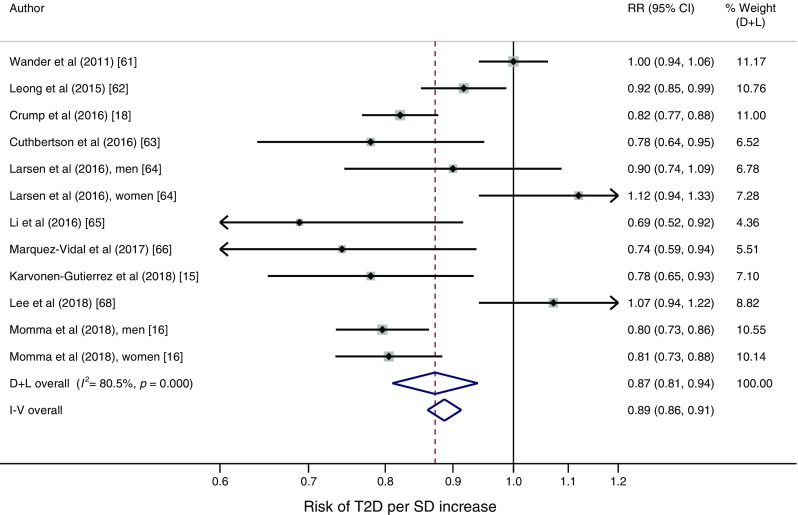
Study-specific RRs per SD increase in muscular strength in models controlling for adiposity. Study weights are from the random-effects analysis (D+L). Pooled RRs from the random-effects analysis (D+L) and the fixed-effects analysis (I-V) are shown based on ten cohorts providing adiposity-controlled estimates. Nine of these cohorts provided per unit estimates (harmonised to per SD) [15, 16, 18, 61, 62, 63, 64, 66, 68] while the linear estimate was modelled using GLST in one study [65]. D+L, DerSimonian and Laird (random-effects model); I-V, inverse variance (fixed-effects model)

In adiposity-controlled models including 39,233 incident cases and 1,713,468 participants, each SD higher muscular strength was associated with a 13% (95% CI 6, 19) lower risk of type 2 diabetes [15, 16, 18, 61–66, 68] (Fig. 4). Pooling the nine available estimates not controlled for adiposity yielded an RR of 0.76 (95% CI 0.64, 0.91) [15, 16, 43, 63–65, 68] per SD higher muscular strength (ESM Fig. 3). Heterogeneity was substantial in both models (*I*^2^ 81% and 91%, respectively). The risk difference associated with a 1 SD higher muscular strength (adiposity-controlled RR) was 142 (95% CI 43, 211) new diabetes cases per 100,000 people per year in the US population aged 45–64 years (additional information in ESM Table 12). Studies applying normalisation of muscular strength to body weight yielded, on average, larger effect sizes (0.83 [95% CI 0.79, 0.86]) than those relying on absolute strength (0.95 [95% CI 0.87, 1.04]).

### Supplementary analyses

Pooled adiposity-controlled estimates were robust to removal of single estimates with RRs ranging from 0.91 to 0.93 for cardiorespiratory fitness and from 0.86 to 0.88 for muscular strength (presented in ESM Tables 13–16). Egger’s test did not indicate a risk of small-study bias in adiposity-controlled estimates (*p* = 0.75 and 0.53) or non-adiposity-controlled estimates for muscular fitness (*p* = 0.83). Some evidence was found in the non-adiposity-controlled cardiorespiratory fitness estimates (*p* = 0.002). The latter appeared to be explained by the influence of one study which had by far the smallest SE and also reported the largest effect size [14]. Funnel plots are presented in ESM Figs. 4–7.

## Discussion

In this systematic review and dose–response meta-analysis of prospective cohort studies we found that cardiorespiratory fitness and muscular strength were inversely associated with the risk of type 2 diabetes. Our results suggest that a 1 MET increase in cardiorespiratory fitness is associated with an 8% lower risk of type 2 diabetes. A 1 SD increase in muscular strength was associated with a 13% lower risk. The magnitude of associations was about twice as large in meta-analysis of study estimates not controlling for adiposity.

The 8% diabetes risk reduction associated with a 1 MET increase is similar to the 10% reduction reported in another recently published meta-analysis on cardiorespiratory fitness [17]. We add robustness to the pooled estimate by including 11 times the number of cases and obtaining additional information or re-analysed data from original study authors. Importantly, we extend the literature by using our updated meta-analysis to estimate hypothetical benefits of population-wide improvements in cardiorespiratory fitness. These calculations inform sound judgement of estimated benefits associated with public health initiatives and are thus essential for decision making. To the best of our knowledge, this is the first quantitative synthesis of the association between muscular strength and risk of type 2 diabetes. Muscle-strengthening activities are included in the WHO physical activity guidelines for adults as an adjunct to aerobic activities [72] and higher handgrip strength is associated with lower mortality [73, 74]. We extend the evidence base supporting higher muscle fitness as an important health marker in middle-aged individuals by showing a lower risk of type 2 diabetes. We note that the RRs for muscular strength varied substantially in magnitude across original studies, ranging from a protective to a detrimental association, while RRs for cardiorespiratory fitness were more consistent. The summary RR for muscular strength should therefore be interpreted with some caution.

Our hypothetical 1 MET increase in the fitness level of the least fit 50% of the population suggested that roughly 4% of type 2 diabetes cases in the population of 40–59-year-olds in the United States could be prevented if this intervention was successfully implemented. Additionally, by modelling the PIF of a population-wide right shift of the cardiorespiratory fitness distribution of US 40–59-year-olds we show that about 21% of new annual type 2 diabetes cases could be prevented if these achieved the same cardiorespiratory fitness distribution as is observed in a same-aged contemporary Western population. Such estimates demonstrate the substantial preventive potential from major structural interventions aiming at increasing cardiorespiratory fitness at the population level. Finally, with an annual (US) incidence of type 2 diabetes in 45–65-year-olds of 1090 cases per 100,000 person-years [37], the risk differences observed with a 1 MET and 1 SD higher fitness level suggest a strong clinical relevance of promoting and monitoring cardiorespiratory fitness and muscular strength as a part of routine primary care. Low population fitness levels are a reflection of insufficient physical activity, with the least fit being most likely to benefit with a change in structured or unstructured activity [10]. PIFs from an intervention in the lowest 50% of the cardiorespiratory fitness distribution thereby provide an estimate of benefits from a feasible intervention target. Importantly, the estimated reductions in type 2 diabetes incidence do not account for any concomitant benefits attributed to the nature of the individual-level intervention that caused fitness to increase. Such interventions include increased levels of physical activity, physical activity-associated weight-loss and/or diet-associated weight-loss.

Cardiorespiratory fitness gains of 1 MET may be achieved by taking up structured exercise for just 4 to 5 months irrespective of age, sex, weight-status and previous commitment to physical activity [8, 12]. Importantly, a 1 MET increase in cardiorespiratory fitness may be achieved in previously inactive individuals by replacing passive with active commuting/transportation such as walking [75] or bicycling [76, 77] for just a few months. A few months of resistance training may produce one-repetition maximum strength gains of 24% in the upper body [9] and substantial strength gains may be achieved across a wide range of ages and glycaemic regulatory capabilities [5, 78, 79]. Despite a strong correlation between handgrip and overall muscular strength [80], it is questionable whether resistance training also translates into changes in maximal handgrip strength [81]. Handgrip strength may thus have more prognostic than interventional utility [74]. As an SD difference in muscular strength corresponded to about one-third of the mean, the absolute changes required to achieve the 13% diabetes risk reduction are also larger than for cardiorespiratory fitness.

Our restricted cubic spline model identified no threshold or levelling off of the cardiorespiratory fitness association but suggested that the type 2 diabetes risk reduction is linear throughout very low to fairly high cardiorespiratory fitness levels. This is not in agreement with observations for all-cause and cardiovascular mortality where relative benefits from cardiovascular fitness gains are much higher at the lower end of the fitness spectrum [27]. We are unable to explain this difference but speculate that it may relate to differences between cardiovascular fitness reflecting overall cardiovascular integrity for cardiovascular disease protection [12] while possibly being more a marker of physical activity in relation to diabetes prevention. On that note, a Mendelian randomisation study found no association between genetic markers of higher handgrip strength and fasting glucose or type 2 diabetes risk [82]. This could suggest that it is engagement in habitual physical activity of sufficient intensity, frequency and duration to increase strength which is biologically relevant and not higher strength per se. Examination of fitness markers and risk of type 2 diabetes stratified by or in combination with physical activity may be informative in future studies. Another interesting avenue of future research would be to investigate potential synergistic effects of combined cardiorespiratory and muscular fitness in relation to diabetes risk [18, 83, 84].

The results from our meta-analyses should be interpreted in the light of at least the following limitations: (1) A number of assumptions and calculations were required to harmonise as much data as possible and the applied assumptions may differ in their accuracy across studies. (2) Muscular strength was predominantly assessed using handgrip strength. It is therefore unknown whether other muscle groups or if other components of muscular fitness (power and endurance) share identical protective associations with type 2 diabetes. However, a cohort of more than one million men found similar associations between handgrip and knee extension strength with all-cause mortality [85]. (3) Despite highly significant between-study heterogeneity most of our stratification parameters made only minor changes to the pooled point-estimates or between-study heterogeneity. Strength cohorts were more heterogeneous with respect to age groups and exposure normalisation than was the case for cardiorespiratory fitness, which is reflected in the forest plots. (4) Cardiorespiratory fitness was routinely presented normalised to body weight (kg). This was not the case for muscular strength. Both of these expressions are likely to be suboptimal as controlling for body weight using kg/kg body weight may induce positive confounding [86] but failure to normalise would result in heavier individuals having, on average, higher (absolute) fitness levels. This could induce negative confounding or even the appearance of higher diabetes risk with increasing fitness. Controlling for BMI is unlikely to ameliorate heterogeneity caused by different normalisation approaches and may in itself represent an overly conservative model because physical activity engagement leading to higher fitness may also help control body weight. On the other hand, there is a consistent gradient of declining BMI with increasing fitness levels which is much larger than what is explained by engagement in physical activity alone [87]. While still incompletely understood, the optimal normalisation procedure to minimise adiposity-induced confounding appears to be normalisation to kilograms of lean body mass [88]. (5) Only one study [53] examined the potential risk of reverse causality bias by excluding from analysis all those developing type 2 diabetes within a few years of follow-up. Future studies should consider this approach. The majority of studies included in meta-analysis followed people for a sufficient time-period to expect that the influence of, for example, undiagnosed or pre-clinical cases should be minimised. (6) As this was a meta-analysis of observational studies we are unable to eliminate the possibility of error from unmeasured or imperfectly assessed confounders and other types of bias. Confounder control varied substantially across studies, but no study succeeded in accounting for all of our pre-determined major putative confounding variables. In addition to the assumption of causal and unbiased effect estimates, our PIF analyses further rely on strong distributional assumptions and should be interpreted cautiously.

In conclusion, our systematic review and meta-analyses provide evidence that higher cardiorespiratory fitness and muscular strength are associated with lower risk of type 2 diabetes. The cardiorespiratory fitness association was linear throughout low to high fitness levels. Physical activities that enhance cardiorespiratory fitness and/or muscular strength should be promoted to decrease risk of type 2 diabetes in individuals and populations.

## Electronic supplementary material

ESM (PDF 1356 kb)

## Data Availability

All data necessary to replicate analyses are presented in the manuscript.

## Abbreviations

D+L: DerSimonian and Laird
FRIEND: Fitness Registry and the Importance of Exercise National Database
GLST: Generalised least squares trend
I-V: Inverse variance
MET: Metabolic equivalent
NOS: Newcastle–Ottawa Scale
PIF: Potential impact fraction
