# Supplementary material for: Cardiorespiratory fitness, muscular strength and risk of type 2 diabetes: a systematic review and meta-analysis
Source: Diabetologia. 2019 Apr 23;62(7):1129–42. doi: 10.1007/s00125-019-4867-4 (PMC6560020; doi:10.1007/s00125-019-4867-4)
Supplement: Supplementary file 1 — (PDF 1356 kb) [file 125_2019_4867_MOESM1_ESM.pdf]

Online-Only Supplemental Material

Cardiorespiratory fitness, muscular strength, and risk of type 2 diabetes: a systematic review and meta-analysis

Jakob Tarp, Andreas Plum Støle, Kim Blond, Anders Grøntved

Table of Contents

ESM Table 1. Search Strategy for PubMed (MEDLINE)

ESM Table 2. Search Strategy for EMBASE

ESM Table 3. Inclusion and exclusion criteria

ESM Table 4. Newcastle-Ottawa quality score of prospective cohort studies of cardiorespiratory fitness and incident type 2 diabetes

ESM Table 5. Newcastle-Ottawa quality score of prospective cohort studies of muscular strength and incident type 2 diabetes

ESM Table 6. List of publications excluded from systematic review because of overlapping information with other cohorts

ESM Table 7. Assumptions, calculations and unpublished data provided by contact with study authors used when harmonizing cardiorespiratory fitness data

ESM Table 8. Assumptions, calculations and unpublished data provided by contact with study authors used when harmonizing muscular strength data

ESM Table 9. Potential impact fractions (PIF) and population attributable fractions (PAF) for counterfactual cardiorespiratory fitness distributions in 40-59-years-old U.S. men and women.

ESM Table 10. Characteristics of studies included in systematic review of cardiorespiratory fitness

ESM Table 11. Characteristics of studies included in systematic review of muscular strength

ESM Table 12. Risk difference associated with a 1-MET increase in cardiorespiratory fitness or a 1-SD increase in muscular strength in age-strata and for the total U.S. adult population

ESM Table 13. Omitting, in turn, one study at a time from linear dose-response meta-analysis of cardiorespiratory fitness estimates including control for adiposity.

ESM Table 14. Omitting, in turn, one study at a time from linear dose-response meta-analysis of cardiorespiratory fitness estimates excluding control for adiposity.

ESM Table 15. Omitting, in turn, one study at a time from linear dose-response meta-analysis of muscular strength estimates including control for adiposity.

ESM Table 16. Omitting, in turn, one study at a time from linear dose-response meta-analysis of muscular strength estimates excluding control for adiposity.

ESM Figure 1. Study-specific relative risks per 1-MET increase in cardiorespiratory fitness in models not controlling for adiposity

ESM Figure 2. Relative risk of type 2 diabetes with increasing cardiorespiratory fitness level modelled using restricted cubic splines using categorical estimates not controlled for adiposity

ESM Figure 3. Study-specific relative risks per standard deviation increase in muscular strength in models not controlling for adiposity

ESM Figure 4. Risk of small-study bias visualized by funnel-plot of cardiorespiratory fitness estimates including control for adiposity.

ESM Figure 5. Risk of small-study bias visualized by funnel-plot of cardiorespiratory fitness estimates excluding control for adiposity.

ESM Figure 6. Risk of small-study bias visualized by funnel-plot of muscular strength estimates including control for adiposity

ESM Figure 7. Risk of small-study bias visualized by funnel-plot of muscular strength estimates excluding control for adiposity

ESM Table 1. Search Strategy for PubMed (MEDLINE)

Exposure	Outcome	Study design
“muscular strength” (ti/ab)	diabetes mellitus, type 2 (mesh)	prospective studies [mesh]
“muscle strength” (ti/ab)	”type II diabetes” (ti/ab)	longitudinal studies [mesh]
muscle strength (MeSH)	”type 2 diabetes” (ti/ab)	observational study [publication type]
”muscle power” (ti/ab)	”diabetes mellitus” (ti/ab)	predic* (ti/ab)
hand strength (MeSH)	diabet* (ti/ab)	Risk (ti/ab)
”grip strength” (ti/ab)		Longitudinal (ti/ab)
”handgrip strength” (ti/ab)		observat* (ti/ab)
”cardiovascular fitness” (ti/ab)		follow-up (ti/ab)
”aerobic fitness” (ti/ab)		cohort (ti/ab)
cardiorespiratory fitness (MeSH)		prospect* (ti/ab)
”cardiorespiratory fitness” (ti/ab)		
physical fitness (mesh)		
”aerobic capacity” (ti/ab)		
”exercise tolerance” (ti/ab)		
”exercise test” (ti/ab)		
“maximal oxygen consumption” (ti/ab)		
“maximal oxygen uptake” (ti/ab)		
vo2max (ti/ab)		
Total hits December 12, 2018: 1951		

ESM Table 2. Search Strategy for EMBASE

Exposure	Outcome	Study design
“muscle strength”.af.	“type 2 diabetes”.af.	“prospective study”.af.
“muscle power”.af.	“type II diabetes”.af.	“observational study”.af.
“hand strength”.af.	“non insulin dependent diabetes mellitus”.af.	“longitudinal study”.af.
“grip strength”.af.	“diabetes mellitus”.af.	“risk factor”.af.
“aerobic fitness”.af.		
“cardiorespiratory fitness”.af.		
“aerobic capacity”.af.		
fitness.af.		
”exercise tolerance”.af.		
“exercise test”.af.		
“maximal oxygen consumption”.af.		
“maximal oxygen uptake”.af.		
vo2max.af.		
“hand strength”.af.		
Total hits December 12, 2018: 2437		

ESM Table 3. Inclusion and exclusion criteria

Component	Inclusion criteria	Exclusion criteria
Population	Studies that include human subjects free of type 2 diabetes at baseline. Cohorts will be included if they consist of participants with conditions that are associated with type 2 diabetes (e.g. obesity, metabolic syndrome, cardiovascular diseases)	Studies not excluding subjects with type 2 diabetes at baseline, studies with a population that consists exclusively of individuals with a chronic disease (e.g. cancer).
Exposure	Cardiorespiratory fitness* assessed by a maximal or sub-maximal stress test of any form Muscular strength** measured as peak score or mean score. Composite scores including >1 unique test will be included. Both isotonic, isometric and isokinetic tests will be included. There are no criteria regarding muscle groups tested. Tests should allow few (<3) repetitions of a task before reaching momentary muscular fatigue	Muscular power*** or endurance****
Outcome	type 2 diabetes	Not type 2 diabetes (also excluding pre-diabetes).
Study design	Cohort studies	Experimental studies, case-control studies, cross-sectional studies, meta-analyses, reviews, reports
Other	Published in English or Scandinavian language. Any publication year	Other languages
For meta-analysis	Results should be provided with relative risk, hazard ratio or odds ratio, and corresponding confidence intervals or information to calculate variance associated with estimates. Estimates should be convertible to the unit size found most appropriate for harmonization	Insufficient information/not possible to convert estimates to chosen unit for harmonization

*Cardiorespiratory fitness is the ability to perform large muscle, dynamic, moderate-vigorous intensity activity for prolonged periods [1]. **Muscular strength is the ability of a muscle to exert maximal force [1]. ***Muscular power is the muscle's ability to exert force per unit of time [1]. ****Muscular endurance is the ability of a muscle to continue to perform without fatigue [1]

ESM Table 4. Newcastle-Ottawa quality score of prospective cohort studies of cardiorespiratory fitness and incident type 2 diabetes

	Study Selection			Comparability of cohorts		Outcome			Stars awarded
	Selection of the non-exposed cohort	Ascertainment of exposure	Demonstration that outcome was not present at start of study	Adiposity	Multivariate adjustment	Assessment of outcome	Length of follow-up	Adequacy of follow up	
Lynch et al., 1996 [2]	A*	A*	A*	A*	B	A*	B	C	5
Katzmarzyk et al., 2007 [3]	A*	C	C	B	B	C	A*	C	2
Sui et al., 2008 [4]	A*	B*	A*	A*	B	A*	A*	B*	7
Carnethon et al., 2009 [5]	A*	B*	A*	A*	B	A*	A*	B*	7
Sieverdes et al., 2010 [6]	A*	B*	A*	A*	B	C	A*	C	5
Skretteberg et al., 2013 [7]	A*	B*	A*	B	B	B*	A*	B*	6
Kuwahara et al., 2014 [8]	A*	C	A*	A*	B	A*	A*	B*	6
Juraschek et al., 2015 [9]	A*	B*	B*	B	B	B*	A*	A*	6
Zaccardi et al., 2015 [10]	A*	A*	A*	A*	B	A*	A*	A*	7
Bantle et al., 2016 [11]	A*	B*	A*	A*	B	A*	A*	C	6
Crump et al., 2016 [12]	A*	B*	B*	A*	B	B*	A*	A*	7
Holtermann et al., 2017 [13]	A*	C	C	A*	B	B*	A*	A*	5
Kokkinos et al., 2017 [14]	A*	B*	B*	A*	B	B*	A*	A*	7

	Selection of the non-exposed cohort	Ascertainment of exposure	Demonstration that outcome was not present at start of study	Adiposity	Multivariate adjustment	Assessment of outcome	Length of follow-up	Adequacy of follow up	**Stars awarded**
Momma et al., 2017 [15]	A*	C	A*	A*	B	A*	A*	B*	6
Kawakami et al., 2018 [16]	A*	C	A*	A*	B	A*	A*	B*	6
Ohlson et al., 1988 [17]	A*	B*	A*	A*	B	A*	A*	B*	7
Williams 2008 [18]	A*	D	D	A*	B	C	A*	C	3
Kinney et al., 2014 [19]	A*	D	D	Unclear	Unclear	C	A*	C	2
Someya et al., 2014 [20]	A*	D	D	A*	B	C	A*	C	3
Jae et al., 2016 [21]	A*	A*	A*	A*	B	A*	A*	C	6
Sydo et al., 2016 [22]	A*	Unclear	D	B	B	B*	A*	A*	4
Wu et al., 2018 [23]	A*	D	A*	A*	B	A*	B	A*	5

We chose not to include the “Representativeness of the exposed cohort” item of the original Newcastle-Ottawa Scale [24] since we find this irrelevant for the evaluation of the internal validity of the studies. Thus, a total of 8 stars were achievable. Study quality reflects assessments in relation to the estimates for which we extracted data and not the study per se.

Newcastle-Ottawa Score key cardiorespiratory fitness

Selection
Selection of the non-exposed cohort
A. Drawn from the same community as the exposed cohort*
B. Drawn from a different source
C. No description of the derivation of the non-exposed cohort

Ascertainment of exposure
A. Directly measured VO2 by gas exchange kinetics to stress-limited max*
B. Treadmill- or ergometry to stress-limited max*
C. Submaximal graded test
D. Other submaximal tests

Demonstration that outcome was not present at start of study
A. Clinical assessment*
B. Medical records, medication status of the patient*
C. Self-report
D. No description

Comparability
Comparability of cohorts on the basis of the design or analysis
A. Study adjusts for BMI or other adiposity index*
B. Study does not adjust for BMI or other adiposity index

Comparability of cohorts on the basis of the design or analysis
A. Study adjusts for (in addition to age, sex and ethnicity if relevant); Muscular fitness, smoking, family history of diabetes, dietary intake (any measure), alcohol consumption, TV-viewing, socioeconomic status (any index) - (4 out of 7)*
B. Study does not adjust for these factors

Outcome
Assessment of outcome
A. Clinical assessment*
B. Medical records, records linkage or medication status of the patient*
C. Self-report
D. No description

Was follow-up long enough for outcomes to occur
A. Yes (> 5 years)*
B. No (< 5 years)

Adequacy of follow up of cohorts
A. Complete follow up (>99%)*
B. Subjects lost to follow up unlikely to introduce bias > 80% subjects followed up or description of those lost suggests unlikely to introduce bias*
C. Follow up rate < 80% and no description of those lost
D. No statement on follow up

ESM Table 5. Newcastle-Ottawa quality score of prospective cohort studies of muscular strength and incident type 2 diabetes

	Study Selection			Comparability of cohorts		Outcome			Stars awarded
	Selection of the non-exposed cohort	Ascertainment of exposure	Demonstration that outcome was not present at start of study	Adiposity	Multivariate adjustment	Assessment of outcome	Length of follow-up	Adequacy of follow up	
Katzmarzyk et al., 2007 [3]	A*	B*	C	B	B	C	A*	C	3
Wander et al., 2011 [25]	A*	B*	A*	A*	B	A*	A*	B*	7
Leong et al., 2015 [26]	A*	B*	C	A*	A*	B*	B	B*	6
Li et al., 2016 [27]	A*	B*	A*	A*	B	A*	B	C	5
Crump et al., 2016 [12]	A*	A*	B*	A*	B	B*	A*	A*	7
Cuthbertson et al., 2016 [28]	A*	B*	C	A*	B	C	A*	C	4
Larsen et al., 2016 [29]	A*	B*	A*	A*	B	A*	A*	C	6
Marquez-Vidal et al., 2017 [30]	A*	B*	A*	A*	B	A*	A*	C	6
Karvonen-Gutierrez et al., 2018 [31]	A*	B*	A*	A*	B	A*	A*	B*	7
Lee et al., 2018 [32]	A*	A*	A*	A*	B	A*	A*	D	6
Momma et al., 2018 [33]	A*	A*	A*	A*	B	A*	A*	B*	7
McGrath et al., 2017 [34]	A*	B*	C	A*	B	C	A*	Unclear	4
Zhang et al., 2018 [35]	A*	B*	A*	Unclear	B	A*	B	B*	5

We chose not to include the "Representativeness of the exposed cohort" item of the original Newcastle-Ottawa Scale [24] since we find this irrelevant for the evaluation of the internal validity of the studies. Thus, a total of 8 stars were achievable. Study quality reflects assessments in relation to the estimates for which we extracted data and not the study per se.

Newcastle-Ottawa Score key muscular strength

Selection

Selection of the non-exposed cohort
A. Drawn from the same community as the exposed cohort*
B. Drawn from a different source
C. No description of the derivation of the non-exposed cohort

Ascertainment of exposure
A. Several major muscle groups measured by dynamometer, 1RM or isokinetic/isometrics/isotonic device*
B. One major muscle groups measured by dynamometer, 1RM or isokinetic/isometrics/isotonic device *
C. No description

Demonstration that outcome was not present at start of study
A. Clinical assessment*
B. Medical records, medication status of the patient*
C. Self-report
D. No description

Comparability

Comparability of cohorts on the basis of the design or analysis
A. Study adjusts for BMI or other adiposity index*
B. Study does not adjust for BMI or other adiposity index

Comparability of cohorts on the basis of the design or analysis
A. Study adjusts for (in addition to age, sex and ethnicity if relevant); Cardiorespiratory fitness, smoking, family history of diabetes, dietary intake (any measure), alcohol consumption, TV-viewing, socioeconomic status (any index) **-** (4 out of 7*
B. Study does not adjust for these factors

Outcome

Assessment of outcome
A. Clinical assessment*
B. Medical records, records linkage or medication status of the patient*
C. Self-report
D. No description

Was follow-up long enough for outcomes to occur
A. Yes (> 5 years)*
B. No (< 5 years)

Adequacy of follow up of cohorts
A. Complete follow up (>99%)*
B. Subjects lost to follow up unlikely to introduce bias > 80% subjects followed up or description of those lost suggests unlikely to introduce bias*
C. Follow up rate < 80% and no description of those lost
D. No statement on follow up

ESM Table 6. List of publications excluded from systematic review because of overlapping information with other cohorts.

**The Coronary Artery Risk Development in Young Adults Study (CARDIA) – Carnethon et al., 2009 [5] & Bantle et al., 2016 [11] included**	
Carnethon et al., 2003 [36]	Fewer cases and shorter follow-up.
**Aerobics Center Longitudinal Study (ACLS) – Sui et al., 2008 [4] & Sieverdes et al., 2010 [6] included**	
Wei et al., 1999 [37]	Fewer cases and participants. Shorter follow-up. Only ascertains cases from clinical assessment.
Le et al., 2008 [38]	Fewer cases and participants. Shorter follow-up. More women included in Sui 2008. Only ascertains cases from clinical assessment.
Lee et al., 2009 [39]	Fewer cases and participants. Shorter follow-up. Only ascertains cases from clinical assessment.
Goodrich et al., 2012 [40]	Fewer participants and shorter follow-up. More women included in Sui 2008. Only ascertains cases from clinical assessment.
Radford et al., 2015 [41]	Fewer cases and participants. Shorter follow-up. More women included in Sui 2008. Only ascertains cases from clinical assessment.
Sloan et al., 2016 [42]	Fewer cases and participants. Shorter follow-up. Only ascertains cases from clinical assessment.
**Tokyo Gas Company Study - Momma et al., 2017 [15] & Kawakami et al 2018 [16] included**	
Sawada et al., 2003 [43]	Fewer cases and participants. Shorter follow-up.
Sawada et al., 2010a [44]	Fewer cases and participants. Shorter follow-up.
Sawada et al., 2010b [45]	Fewer cases and participants. Shorter follow-up.
Kawakami et al., 2014 [46]	Fewer cases and participants. Shorter follow-up.
Sloan et al., 2018 [47]	Fewer cases and participants. Shorter follow-up.
**Veterans Affairs Medical Center Study - Kokkinos et al., 2017 [14] included**	
Narayan et al., 2016 [48]	Conference abstract.
**Oslo Ischemia Study – Skretteberg et al., 2013 [7] included**	
Bjørnholt et al., 2001 [49]	Fewer cases and participants. Shorter follow-up.

ESM Table 7. Assumptions, calculations and unpublished data provided by contact with study authors used when harmonizing cardiorespiratory fitness data.

<table>
<tr><th>Author</th><th>Assumptions/calculations</th></tr>
<tr><td>Lynch et al., 1996 [2]</td><td>Categorical data:
– Individuals in quartiles of CRF unclear
○ Assigned 187 individuals to most fit quartile, 187 to other quartiles
– MET-level in quartiles unclear
○ Assigned dose as mid-point (e.g. 28.35 for category 25.8 to 31.0) for two middle categories
○ Assigned doses as 2.55 from upper/lower cut-off in outer categories
○ Divided oxygen uptake in ml/kg/min by 3.5</td></tr>
<tr><td>Zaccardi et al., 2015 [10]</td><td>No transformations applied</td></tr>
<tr><td>Katzmarzyk et al., 2007 [3]</td><td>Linear models:
– Estimate presented per standard deviation increase in CRF
○ Calculated pooled standard deviation of CRF*
○ Converted estimate in standard deviations to per 1-MET [50]</td></tr>
<tr><td>Sui et al., 2008 [4]</td><td>Categorical data:
– MET-level in tertiles unclear
○ Pooled “No diabetes” and “diabetes” MET-values in Table 1* and assigned this value as METs-values in middle tertile
○ Assumed normal-distribution of CRF and subtracted/added 1 MET for dose in outer tertiles under the assumption that the standard deviation of CRF was 2 METs [51]
Linear models:
○ GLST applied on categorical estimates</td></tr>
<tr><td>Sieverdes et al., 2010 [6]</td><td>Data provided by personal communication
<table><tr><th>Group</th><th>Cases</th><th>Total participants</th><th>Person-years</th><th>Dose (METs)</th></tr><tr><td>Ref</td><td>307</td><td>5754</td><td>114,320</td><td>9.0</td></tr><tr><td>1</td><td>142</td><td>5718</td><td>107,293</td><td>11.3</td></tr><tr><td>2</td><td>94</td><td>5900</td><td>101,551</td><td>12.6</td></tr><tr><td>3</td><td>46</td><td>6072</td><td>101,196</td><td>14.4</td></tr></table>Linear models:
○ GLST applied on categorical estimates</td></tr>
</table>

<table>
<tr><th>Author</th><th>Assumptions/calculations</th></tr>
<tr><td>Carnethon et al., 2009 [5]</td><td>Linear models:
– Estimates presented per standard deviation decrease in treadmill-time
    o Calculated sex-ethnicity specific METs [52] associated with a 1 standard deviation (using sex-ethnicity based standard deviation) increase in treadmill time using the difference in METs from the mean treadmill time to mean + 1 standard deviation of treadmill time based on reported data.
    o Convert sex-ethnicity specific estimate to per 1-MET [50]
    o Invert estimate from decrease to increase CRF by: exponentiate(-log(estimate))
    o Using fixed-effects meta-analysis to pool ethnicity-stratified data</td></tr>
<tr><td>Skretteberg et al., 2013 [7]</td><td>Linear models:
– Estimate presented per standard deviation increase in CRF
    o Assumed standard deviation of 2 METs [51]
    o Converted estimate in standard deviations to per 1-MET [50]</td></tr>
<tr><td>Kuwahara et al., 2014 [8]</td><td>Data provided by personal communication
<table>
<tr><th>Group</th><th>Cases</th><th>Total participants</th><th>Person-years</th><th>Dose (METs)</th></tr>
<tr><td>Ref</td><td>65</td><td>886</td><td>5084</td><td>9.0</td></tr>
<tr><td>1</td><td>56</td><td>890</td><td>5378</td><td>10.2</td></tr>
<tr><td>2</td><td>45</td><td>875</td><td>5346</td><td>11.4</td></tr>
<tr><td>3</td><td>33</td><td>872</td><td>5379</td><td>13.1</td></tr>
</table>
Linear models:
    o GLST applied on categorical estimates</td></tr>
<tr><td>Juraschek et al., 2015 [9]</td><td>Categorical models:
BMI-adjusted models
– Cases in four CRF categories unclear
    o Estimated cases based on unadjusted 5-year unadjusted cumulative incidence scaled to match total diabetes incidence (from low-fit; 1296, 2330, 2396, 828).

Excluding BMI from models
– Total participants and cases in four CRF categories unclear
    o Calculated total participants and cases based on assumption of identical distribution of participants and cases as in full cohort (participants: from low-fit; 1290, 2898, 4471, 3091. Cases: from low-fit; 324, 583, 599, 207).</td></tr>
<tr><td>Bantle et al., 2016 [11]</td><td>Categorical data:
– MET-level in tertiles unclear
    o Calculated MET from time on treadmill using CARDIA formula [52]
– Diabetes cases in tertiles unclear
    o Data provided by personal communication (from low-fit; 204, 105, 84).
Linear models:
    o GLST applied on categorical estimates</td></tr>
</table>

<table>
<tr><th>Author</th><th>Assumptions/calculations</th></tr>
<tr><td>Crump et al., 2016 [12]</td><td>Categorical data:
– MET-level in tertiles unclear
o Median watt/kg in tertiles provided by personal communication (from low-fit; 3.21, 3.84, 4.62)
o Estimated METs in tertiles from watt/kg by formula: ml O_2/min/kg = 8.0697 x watt/kg + 9.042817
▪ Formula derived by (unpublished) linear regression of maximal oxygen uptake on watt/kg in 278 Danish men aged 20-28 years from the general population participating in the European Youth Heart Study [53]. Watt/kg explained 71% of the variance in maximal oxygen uptake as measured by indirect calorimetry
o Divided maximum oxygen uptake by 3.5
– Reference group is most fit tertile
o Converted reference group to least fit tertile by Hamling-method implemented in Microsoft Excel macro [54]

Linear models:
– Estimate presented per 1 watt/kg
o Estimated per MET from watt/kg by formula: ml O_2/min/kg) = 8.0697 x watt/kg + 9.042817
o Converted estimate to per 1-MET [50]</td></tr>
<tr><td>Holtermann et al., 2017 [13]</td><td>Data provided by personal communication

Group | Cases | Total participants | Person-years | Dose (METs)
Ref | 178 | 1389 | 34,531 | 7.1
1 | 137 | 1181 | 32,904 | 8.6
2 | 102 | 1226 | 35,154 | 10.0
3 | 101 | 1192 | 37,008 | 12.0

Categorical data:
– MET-level in tertiles unclear
o Divided oxygen uptake in ml/kg/min by 3.5
Linear models:
BMI-adjusted:
– Estimates presented per 10 ml O_2 /kg/min
o Divided by 3.5 to obtain estimate in METs
o Converted estimate in standard deviations to per 1-MET [50]

Excluding BMI from models
o GLST applied on categorical estimates</td></tr>
</table>

Author	Assumptions/calculations
Kokkinos et al., 2017 [14]	Data provided by personal communication<br><br>Group / Cases / Total participants / Person-years / Dose (METs)<br>Ref / 336 / 954 / 15,915 / 7.8<br>1 / 328 / 1201 / 17,713 / 8.3<br>2 / 288 / 1242 / 18,529 / 9.2<br>3 / 123 / 695 / 14,881 / 11.2<br><br>Linear models:<br>○ GLST applied on categorical estimates
Momma et al., 2017 [15]	Categorical data:<br>– MET-level in quartiles unclear<br>○ Divided oxygen uptake in ml/kg/min by 3.5<br><br>Linear models:<br>○ GLST applied on categorical estimates
Kawakami et al., 2018 [16]	Categorical data:<br>– MET-level in quartiles unclear<br>○ Divided oxygen uptake in ml/kg/min by 3.5<br><br>Linear models:<br>○ GLST applied on categorical estimates

*Using formula provided by the Cochrane Handbook for Systematic Reviews of Interventions, table 7.7.a [55]. CRF; cardiorespiratory fitness, MET; metabolic equivalent, GLST; generalized least-squares trend-estimation, CARDIA; Coronary Artery Risk Development in Young Adults

ESM Table 8. Assumptions, calculations and unpublished data provided by contact with study authors used when harmonizing muscular strength data

<table>
<tr><th>Author</th><th>Models and assumptions/calculations</th></tr>
<tr><td>Katzmarzyk et al., 2007 [3]</td><td>No transformations applied</td></tr>
<tr><td>Wander et al., 2011 [25]</td><td>– Results presented per 10 pounds increase in muscular strength
o Assumed variance estimate in table 1 are standard error of the mean
o Calculated pooled standard deviation of muscular strength from table 1*
o Converted estimates to per standard deviation increase [50]</td></tr>
<tr><td>Leong et al., 2015 [26]</td><td>– Results presented per 5 kg decrease in muscular strength
o Assumed identical standard deviation as reported in table 1 in sample excluding individuals with prevalent cancer and cardiovascular disease
o Converted estimates to per standard deviation increase [50]
o Invert estimate from decrease to increase in muscular strength using: exponentiate(-log(estimate))</td></tr>
<tr><td>Li et al., 2016 [27]</td><td>Data provided by personal communication
<table>
<tr><th>Group</th><th>Cases</th><th>Total participants</th><th>Person-years</th><th>Dose (kg / kg body-weight)</th></tr>
<tr><td>Ref</td><td>63</td><td>408</td><td>1893</td><td>0.43</td></tr>
<tr><td>1</td><td>37</td><td>408</td><td>1920</td><td>0.54</td></tr>
<tr><td>2</td><td>29</td><td>408</td><td>1946</td><td>0.62</td></tr>
<tr><td>3</td><td>17</td><td>408</td><td>1977</td><td>0.72</td></tr>
</table>
o GLST applied on categorical estimates
o Converted estimates to per standard deviation increase [50]</td></tr>
<tr><td>Crump et al., 2016 [12]</td><td>– Results presented per 1 N/kg body-weight increase in composite muscular strength score
o Median Newtons/kg body-weight in tertiles provided by personal communication (from low-fit; 25.33, 30.17, 34.03)
o Calculated pooled standard deviation of muscular strength from table 1*
o Converted estimates to per standard deviation increase [50]
o Moved upper confidence limit from 0.97 to 0.98 to achieve symmetry around point-estimate</td></tr>
<tr><td>Cuthbertson et al., 2016 [28]</td><td>Data provided by personal communication</td></tr>
<tr><td>Larsen et al. 2016 [29]</td><td>No transformations applied</td></tr>
</table>

Author	Assumptions/calculations
Marques-Vidal et al., 2017 [30]	– Results presented per 5 kg increase in muscular strength<br>○ Calculated pooled standard deviation of muscular strength from table 1*<br>○ Converted estimates to per standard deviation increase [50]
Karvonen-Gutierrez et al., 2018 [31]	– Results presented per 0.1 kg/kg body-weight increase in muscular strength<br>○ Converted estimates to per standard deviation increase [50]
Lee et al., 2018 [32]	Data provided by personal communication
Momma et al., 2018 [33]	Data provided by personal communication

*Using formula provided by the Cochrane Handbook for Systematic Reviews of Interventions, table 7.7.a [55]

ESM Table 9. Potential impact fractions (PIF) and population attributable fractions (PAF) for counterfactual cardiorespiratory fitness distributions in 40-59-years-old U.S. men and women.

Intervention	Sex	Observed CRF distribution [56]	RR per 1-MET	Counterfactual CRF distribution	PIF
1-MET CRF increase achieved in the least fit 50%	Men	FRIEND database (US)[a] Mean: 10.37 SD: 2.76	0.80 (non-adiposity-controlled)	Mean: 10.82 SD: 2.38	13.4%
1-MET CRF increase achieved in the least fit 50%	Women	FRIEND database (US)[a] Mean: 7.45 SD: 2.05	0.80 (non-adiposity-controlled)	Mean: 7.86 SD: 1.68	11.3%
1-MET CRF increase achieved irrespective of initial CRF	Men	FRIEND database (US)[a] Mean: 10.37 SD: 2.76	0.80 (non-adiposity-controlled)	Mean:11.37 SD: 2.76	19.7%
1-MET CRF increase achieved irrespective of initial CRF	Women	FRIEND database (US)[a] Mean: 7.45 SD: 2.05	0.80 (non-adiposity-controlled)	Mean: 8.45 SD: 2.05	19.5%
Achieve same CRF distribution as age-matched Norwegian population-based sample[b]	Men	FRIEND database (US)[a] Mean: 10.37 SD: 2.76	0.80 (non-adiposity-controlled)	Norwegian HUNT study [57] (men aged 40-59 years) Mean: 12.69 SD: 2.31	43.4%
Achieve same CRF distribution as age-matched Norwegian population-based sample[b]	Women	FRIEND database (US)[a] Mean: 7.45 SD: 2.05	0.80 (non-adiposity-controlled)	Norwegian HUNT study [57] (women aged 40-59 years) Mean: 10.24 SD:1.92	46.6%
Achieve same CRF distribution as most active tertile of age-matched individuals from a Norwegian population-based sample[c]	Men	FRIEND database (US)[a] Mean: 10.37 SD: 2.76	0.80 (non-adiposity-controlled)	Norwegian HUNT study [57] (men aged 40-59 years) Mean: 14.09 SD: 2.31	58.4

Achieve same CRF distribution as most active tertile of age-matched individuals from a Norwegian population-based sample[c]	Women	FRIEND database (US)[a] Mean: 7.45 SD: 2.05	0.80 (non-adiposity-controlled)	Norwegian HUNT study [57] (women aged 40-59 years) Mean: 11.19 SD: 2.08	55.9
Elimination of “unfit” category (bottom 25% of CRF)	Men	FRIEND database (US)[a] Mean: 10.45 SD: 2.77	0.80 (non-adiposity-controlled)	-	PAF[d] 15.3 %
Elimination of “unfit” category (bottom 25% of CRF)	Women	FRIEND database (US)[a] Mean: 7.45 SD: 2.05	0.80 (adiposity-controlled)	-	PAF[d] 11.4 %

[a]Age-groups combined via The Cochrane Collaboration. Higgins J & Green S (Editors). Cochrane Handbook for Systematic Reviews of Interventions. Table 7.7.a: Formulae for combining groups [55]. [b]"Feasible minimum risk”. [c]"Plausible minimum risk”. [d]PAFs [58] for low cardiorespiratory fitness were calculated by defining the bottom 25% of the population CRF distribution as unfit (<8.4 METs would be classified as unfit for men whereas women with a CRF <6.0 METs would be classified as unfit) based on the U.S. FRIEND database at 40-59 years of age. We then estimated the proportion of total diabetes cases which could theoretically be prevented by changing the cardiorespiratory fitness level of all unfit adults to the fitness level matching the distribution of the population of “fit” individuals (≥25th percentile). RR’s were based on a contrast between the fitness level of the sex-specific 12.5th percentile (the midpoint of the 1st to 25th percentile interval) and the 62.5th percentile (the midpoint of the 25th to 99th percentile) estimated from the restricted cubic spline model. This analysis is comparable to conventional PAF calculations based on eliminating the exposure and “shifting” exposed individuals into matching the distribution of the “non-exposed” reference category (above the sex-specific MET cut-points as specified above). As the PIF is calculated based on a distributional change, rather than complete elimination, it may be preferable over PAFs in the case of a continuous exposure were the minimum risk is achieved at a non-zero exposure level [59]. CRF; cardiorespiratory fitness, PIF; potential impact fraction, PAF; population attributable fraction.

ESM Table 10. Characteristics of studies included in systematic review of cardiorespiratory fitness

Study	Country (study name)	Numbers analysed, description and recruitment period of cohort	Men (%) Ethnicity (%)	Age at baseline (years)	Follow-up (years)	Outcome assessment	Cumulative type 2 diabetes incidence	CRF assessment	Estimates from manuscript used in meta-analysis (RR/OR/HR with 95% CI)	Model control
Lynch et al., 1996 [2]	Finland<br><br>Kuopio Ischemic Heart Disease Risk Factor Study	751<br><br>Population-based random sample (78.1 % consenting to study) of men from the town of Kuopio, Finland<br><br>1984-1989	100%<br><br>Caucasian	Mean (SD): 51.2 (6.7)	Median: 4.2<br><br>Range: 3.8 – 5.2	Clinical assessment	5.2 %<br><br>39 / 751	Maximal graded exercise test on bicycle ergometer	OR relative to least fit quartile<br><br>Multivariable + BMI<br>1<br>0.77 (0.32 – 1.85)<br>0.26 (0.08 – 0.82)<br>0.15 (0.03 – 0.79)	Age, baseline FPG, triglyceride, systolic BP, parental history of diabetes, alcohol consumption, BMI
Zaccardi et al., 2015 [10]	Finland<br><br>Kuopio Ischemic Heart Disease Risk Factor Study	2520<br><br>Population-based random sample (78.1 % consenting to study) of men from the town of Kuopio, Finland<br><br>1984-1989	100%<br><br>Caucasian	Mean (SD): 53.0 (5.2)<br><br>Range: 42-60	Median (IQR): 23 (18 – 25)	Clinical assessment + records linkage	6.1 %<br><br>153 / 2520	Maximal graded exercise test on bicycle ergometer	HR per 1-MET increase<br>Multivariable + BMI<br>0.95 (0.86 – 1.04)	Age, systolic BP, HDL-c, family history of diabetes, smoking, education, socioeconomic status, BMI
Katzmarzyk et al., 2007 [3]	Canada<br><br>Canadian Physical Activity Longitudinal Study	852<br><br>Participants in the Canada Fitness Survey and/or Campbell's Survey of Well-being in Canada. Sampled to be	46 %<br><br>Caucasian	Mean (SD): 37.1 (12.2)<br><br>Range: 18 – 69	Mean: 15.5	Self-report	5.0 %<br><br>43 / 852<br><br>(calculated based on assumption of identical incidence in sample with data)	Sub-maximal graded step-test (modified Canadian Aerobic Fitness Test)	OR per SD increase<br><br>Multivariable - BMI<br>0.30 (0.14 – 0.60)	Age, sex, smoking, alcohol intake, parental history of diabetes

		representative of the Canadian population<br><br>1988								
Sui et al., 2008 [4]	USA<br><br>Aerobics Center Longitudinal study	6249<br><br>Women participating in a preventive medical evaluation at the Cooper Clinic, Texas.<br><br>1971 - 2004	0 %<br><br>Caucasian	Mean (SD): 43.8 (10.0)<br><br>Range: 20 - 79	Up to 17	Self-report + Clinical assessment	2.3 %<br><br>(143 / 6249)	Maximal graded treadmill test after modified Balke protocol	HR relative to least fit tertile according to age-specific distributions of treadmill time<br><br>Multivariable - BMI<br>1<br>0.76 (0.52 – 1.11)<br>0.49 (0.31 – 0.77)<br><br>Multivariable + BMI<br>1<br>0.86 (0.59 – 1.25)<br>0.61 (0.38 – 0.96)	Age, smoking, alcohol intake, hypertension, family history of diabetes, survey-response pattern, BMI
Sieverdes et al., 2010 [6]	USA<br><br>Aerobics Center Longitudinal study	23,444<br><br>Men participating in a preventive medical evaluation at the Cooper Clinic, Texas.<br><br>1970-2003	100%<br><br>Caucasian	Mean (SD): 45 (9.8)<br><br>Range: 20 - 85	19 (median)	Self-report	2.5%<br><br>589 / 23,444	Maximal graded treadmill test after modified Balke protocol	HR relative to least fit quartile (additional estimates provided following personal communication)<br><br>Multivariable - BMI<br>1<br>0.51 (0.40 – 0.64)<br>0.38 (0.29 – 0.51)<br>0.17 (0.12 – 0.25)<br><br>Multivariable + BMI<br>1<br>0.66 (0.52 – 0.84)<br>0.56 (0.42 – 0.75)<br>0.29 (0.20 – 0.44)	Age, examination year, survey response pattern, physical activity, smoking, alcohol consumption, hypercholesterolemia, hypertension, family history of diabetes, family history of CVD, BMI
Carnethon et al., 2009 [5]	USA<br><br>Coronary Artery Risk Development in Young	3989<br><br>Recruitment aimed to obtain a representative sample of	46 %<br><br>54% Caucasian<br><br>46%	Mean: 24.9<br><br>Range: 18 - 30	Up to 20	Clinical assessment	6.8 %<br><br>271 / 3989	Maximal graded treadmill test after modified Balke protocol	HR per SD increase in treadmill time<br><br>Multivariable - BMI<br>*White men*<br>3.36 (2.44 – 4.63)<br>*Black men*	Age, smoking, family history of diabetes, fasting glucose

	Adults (CARDIA)	population in four communities<br><br>1985 - 1986	Black						1.80 (1.26 – 2.58)<br>*White women*<br>3.15 (2.03 – 4.87)<br>*Black women*<br>2.03 (1.41 – 2.91)	
Bantle et al., 2016 [11]	USA<br><br>Coronary Artery Risk Developm ent in Young Adults (CARDIA)	3358<br><br>Recruitment aimed to obtain a representative sample of population in four communities<br><br>1985 - 1986	44 %<br><br>53% Caucasia n<br>47% Black	Mean (SD):<br>25.0 (3.6)<br><br>Range:<br>18 - 30	25	Clinical assessment	11.7 %<br><br>393 / 3358	Maximal graded treadmill test after modified Balke protocol	OR relative to least fit tertile<br><br>Multivariable + BMI<br>1<br>1.06 (0.88 – 1.27)<br>0.62 (0.49 – 0.79)	Age, sex, ethnicity, field-center, physical activity, education, smoking, energy intake, diet-quality, BMI
Skretteberg et al., 2013 [7]	Norway<br><br>Oslo Ischemia Study	1662<br><br>Healthy men of five governmental agencies in Oslo<br><br>1972-1975	100 %<br><br>Caucasia n	Approx mean (SD):<br>50 (5.5)<br><br>Range:<br>40 - 59	Median:<br>28.5<br><br>Range:<br>0.3 – 34.3	Records linkage	12.1 %<br><br>202 / 1662	Maximal graded exercise test on bicycle ergometer	HR per SD increase<br><br>Multivariable - BMI<br>0.71 (0.58 – 0.86)	Age, fasting whole-blood glucose, family history of maternal diabetes
Kuwahara et al., 2014 [8]	Japan<br><br>Japan Epidemiolo gy Collaborati on on Occupatio nal Health	3523<br><br>Employees at a company in Japan participating in an annual health-examination<br><br>2003-2005	100 %<br><br>Asian	Mean (SD):<br>42.2 (10.4)<br><br>Range:<br>18-61	Mean:<br>6.0	Clinical assessment	5.6 %<br><br>199 / 3523	Sub-maximal graded exercise test on bicycle ergometer	HR relative to least fit quartile (additional estimates provided following personal communication)<br><br>Multivariable - BMI<br>1<br>0.94 (0.65 – 1.35)<br>0.80 (0.54 – 1.17)<br>0.64 (0.42 – 0.99)<br><br>Multivariable + BMI<br>1<br>1.10 (0.76 – 1.59)<br>1.03 (0.69 – 1.54)<br>0.95 (0.60 – 1.50)	Age, baseline year, smoking, alcohol consumption, sleep duration, family history of diabetes, hypertension, BMI
Juraschek et al., 2015 [9]	USA	46,979	52 %	Mean (SD):	Median (IQR)	Records linkage	14.6 %	Maximal graded	HR relative to least fit of four groups	Age, sex, ethnicity, history of

	The FIT (Henry Ford Exercise Testing) project)	(11,750 in BMI-subsample)<br><br>Patients referred to exercise stress-test at Henry Ford Health System Affiliated Subsidiaries in Detroit<br><br>1991-2009	66 % Caucasian<br>27 % Black<br>7 % Other	52.5 (12.6)	5.2 (2.6-8.3)		6851 / 46,979<br><br>Assumed identical incidence in BMI-subsample	treadmill test (Bruce protocol).	based on distribution of data<br><br>Multivariable - BMI<br>1<br>0.96 (0.89 – 1.03)<br>0.77 (0.71 – 0.83)<br>0.46 (0.41 – 0.51)<br><br>Multivariable + BMI<br>1<br>0.99 (0.88 – 1.11)<br>0.90 (0.79 – 1.02)<br>0.64 (0.54 – 0.75)<br><br>RR per 1-MET increase<br>Multivariable - BMI<br>0.92 (0.91 – 0.93)<br><br>RR per 1-MET increase<br>Multivariable + BMI<br>0.96 (0.94 – 0.97)	hypertension, hypertension medication use, ACE inhibitor use, ARB use, β-blocker use, diuretic use, history of hyperlipidemia, lipid-lowering medication use, statin use, history of obesity, family history of CHD, smoking, physical activity, pulmonary disease medication use, depression medication use, indication for stress testing<br>+<br>BMI in sub-sample
Crump et al., 2016 [12]	Sweden<br><br>Swedish Military Conscription Registry Study	1,534,425<br><br>Men participating in military conscription examination (97-98% of Swedish men)<br><br>1969 - 1997	100%<br><br>Caucasian	All 18	Mean: 25.7<br><br>Up to 40	Records linkage	2.2 %<br><br>34,008 / 1,534,425	Maximal exercise test on bicycle ergometer	HR relative to most fit tertile<br><br>Multivariable + BMI<br>1<br>1.15 (1.11 – 1.20)<br>1.72 (1.65 – 1.79)<br><br>Multivariable + BMI<br>0.65 (0.64 – 0.67)	Year of military conscription examination, muscular strength, family history of diabetes, education, neighbourhood socioeconomic status, BMI
Holtermann et al., 2017 [13]	Denmark<br><br>Copenhagen Male Study	4988<br><br>Employees at 14 workplaces<br><br>1970-1971	100 %<br><br>Caucasian	Mean (SD): 48.7 (5.4)	Mean (SD): 28.0 (11.2)<br><br>Up to 44	Records linkage	10.4 %<br><br>518 / 4988	Sub-maximal graded exercise test on bicycle ergometer	HR relative to least fit quartile (additional estimates provided following personal communication)<br><br>Multivariable - BMI<br>1<br>0.83 (0.66 – 1.05)<br>0.61 (0.47 – 0.78)	Age, smoking, status, grams of tobacco per day, systolic BP, diastolic BP, physical activity, alcohol consumption, social class, BMI

									0.57 (0.43 – 0.74)<br><br>Multivariable + BMI<br>1<br>0.90 (0.72 – 1.13)<br>0.74 (0.57 – 0.96)<br>0.75 (0.57 – 0.98)<br><br>Per 10 ml O_2 kg/min<br>0.86 (0.75 – 0.98)	
Momma et al., 2017 [15]	Japan<br><br>Tokyo Gas Company	7158<br><br>Employees at Tokyo Gas Company participating in law-required health-examinations<br><br>1986	100 %<br><br>Asian	Median (IQR):<br>37 (32 - 45)<br><br>Range:<br>20 - 60	Range:<br>18 - 23	Clinical assessment	20.9 %<br><br>1495 / 7158	Sub-maximal graded exercise test on bicycle ergometer	HR relative to least fit quartile<br><br>Multivariable + BMI<br>1<br>0.81 (0.71 – 0.93)<br>0.81 (0.70 – 0.93)<br>0.64 (0.54 – 0.75)	Age, systolic BP, family history of diabetes, smoking, alcohol intake, desk work, frequency of CRF measurement, BMI
Kawakami et al., 2018 [16]	Japan<br><br>Tokyo Gas Company	7804<br><br>Employees at Tokyo Gas Company participating in law-required health-examinations<br><br>1986	100 %<br><br>Asian	Mean (SD):<br>38 (10)<br><br>Range:<br>19-60	Median:<br>19<br><br>Up to 23	Clinical assessment	13.4 %<br><br>1047 / 7804	Sub-maximal graded exercise test on bicycle ergometer	HR relative to least fit quartile<br><br>Multivariable - BMI<br>1<br>0.78 (0.67 – 0.91)<br>0.63 (0.54 – 0.75)<br>0.43 (0.35 – 0.52)	Age, systolic BP, family history of diabetes, smoking, alcohol intake
Kokkinos et al., 2017 [14]	USA<br><br>Veterans Affairs Medical Centers study	4092<br><br>Veterans participating in the ETHOS or VETS studies who are treated with statins<br><br>1986-2014	96 %<br><br>34 % Caucasian<br>66 % Black	Mean (SD):<br>59 (10.8)	Mean (SD):<br>8.3 (5.2)	Records Linkage	26.2 %<br><br>1075 / 4092	Maximal graded treadmill test (Bruce protocol) or individualized ramp protocol	HR relative to least fit quartile (additional estimates provided following personal communication)<br><br>Multivariable - BMI<br>1<br>0.77 (0.66 – 0.90)<br>0.67 (0.57 – 0.79)<br>0.55 (0.45 – 0.68)	Age, ethnicity, sex, β-blockers, calcium channel blockers, diuretics, ACE inhibitor use, ARB use, smoking, hypertension, sleep apnea, alcohol/drug abuse, BMI

									Multivariable + BMI 1<br>0.82 (0.70 – 0.95)<br>0.76 (0.65 – 0.90)<br>0.66 (0.53 – 0.82)	
Ohlson et al., 1988 [17]	Sweden<br><br>Gothenburg Male Population study	766<br><br>Individuals born in 1913 with date of birth divisible by 3 and living in the city of Gothenburg (88 % of invited participating in study I 1963, 94 % of these agreeing in 1967)<br><br>1967	100 %<br><br>Caucasian	Mean: 54	13.5	Clinical assessment	6.1 %<br><br>47 / 766	Maximal graded exercise test on bicycle ergometer	*Data not harmonizable for inclusion in meta-analysis*<br><br>No significant association found (data not reported)	Unclear
Williams 2008 [18]	USA<br><br>National Runners' Health Study	33,574<br><br>Subscribers to a running magazine and participants in running races in the US (approx. 15 % of targeted individuals participating in study)<br><br>1991 - 1994	73 %<br><br>Ethnicity not stated	Approx Mean (SD): 43.1 (10.7)	Approx Mean: 7.6	Self-report	Men:<br>0.68 %<br><br>197 / 24,517<br><br>Women:<br>0.23 %<br><br>28 / 9057	Self-reported best 10-km race during previous 5 year	*Data not harmonizable for inclusion in meta-analysis*<br>OR per m/s<br><br>Multivariable - BMI<br>0.23 (0.16 – 0.33)<br><br>Multivariable + BMI + BMI-squared<br>0.46 (0.31 – 0.67)	Age, follow-up time, intake of red meat, fish, fruit, alcohol intake, physical activity (running distance/week), BMI, BMI-squared
Kinney et al., 2014 (abstract only) [19]	USA<br><br>COPD Genetic epidemiology study	7080<br><br>Smokers with and without chronic obstructive	Unclear	Unclear	Approx Mean: 3.2	Unclear	5.5 %<br><br>392 / 7080	6 Minute Walk Distance	*Data not harmonizable for inclusion in meta-analysis*<br>HR per 100 feet lower walk distance<br>0.94 (0.91 – 0.97)	Unclear

		pulmonary disease<br><br>2008-2011								
Someya et al., 2014 [20]	Japan<br><br>Department of Physical Education Juntendo University Study	570<br><br>Male alumni at the Department of Physical Education Juntendo University<br><br>1971-1991	100 %<br><br>Asian	Approx median : 23	Median (IQR):<br>26<br>(45 – 52)	Self-report	3.9 %<br><br>22 / 579	1500 meters endurance run	*Data not harmonizable for inclusion in meta-analysis*<br>HR relative to least fit tertile<br><br>Multivariable + BMI<br>1<br>0.40 (0.14 – 1.13)<br>0.26 (0.07 – 1.00)	Age, year of graduation, smoking, college sports-club participation, BMI
Jae et al., 2016 [21]	South Korea<br><br>Samsung Medical Center Study	3770<br><br>Participants in two health-examinations at Samsung Medical Center, Seoul<br><br>1998-2008	100 %<br><br>Asian	Mean:<br>47<br><br>Range:<br>20 - 76	Median:<br>5.0<br><br>Range:<br>1-12	Clinical assessment	4.5 %<br><br>170 / 3770	Maximal graded treadmill test (Bruce protocol)	*Data not harmonizable for inclusion in meta-analysis*<br>RR relative to least fit 50%<br><br>Multivariable - BMI<br>1<br>0.70 (0.51 – 0.97)<br><br>Multivariable + BMI<br>1<br>0.75 (0.54 – 1.05)	Age, FPG, systolic BP, total cholesterol, HDL-c, LDL-c, triglycerides, uric acid, resting heart rate, smoking, alcohol intake, BMI
Sydo et al., 2016 (abstract only) [22]	USA<br><br>Mayo Clinic Study of Past Smokers	7090<br><br>Past smokers with an exercise test from the Mayo Clinic, Rochester<br><br>1993 - 2010	67 %<br><br>Unclear	Mean (SD):<br>54 (11)	Mean (SD):<br>12 (5)	Records linkage	8.0 %<br><br>567 / 7090	“Exercise test”	*Data not harmonizable for inclusion in meta-analysis*<br>Difference in rates in three groups of<br><80 % FAC (ref)<br>80-100 % FAC<br>>100 % FAC<br><br><80% FAC:<br>14 %<br><br>80-100% FAC:<br>6 %, p<0.01	Age, sex

									>100 % FAC<br>4%, p=0.01	
Wu et al., 2018 [23]	Taiwan<br><br>Taiwan Armed Forces Study	27,287<br><br>Member of Taiwan military forces without severe chronic medical conditions or disability participating in annual compulsory health examinations	85 %<br><br>Asian	Mean (SD):<br>33 (6)	All 2	Clinical assessment	Unclear	3000 meters endurance run	*Data not harmonizable for inclusion in meta-analysis*<br>Significant association observed for men without MetS only. No significant association for men with MetS or for women irrespective of MetS status	Age, aspartate transaminase, serum uric acid, hemoglobin, serum creatine, proteinuria, family history of cardiovascular disease, smoking, alcohol consumption, betel nut chewing, BMI

Abbreviations: CRF; cardiorespiratory fitness, RR; relative risk, OR; odds ratio, HR; hazard ratio, SD; standard deviation, BMI; body-mass index, CVD; cardiovascular disease, IQR; inter-quartile range, BP; blood pressure, ACE: angiotensin-converting enzyme inhibitor. ARB: angiotensin II- receptor blocker, CHD; coronary heart disease, MET; metabolic equivalent, FPG; fasting plasma glucose, HDL-c; high-density-lipoprotein cholesterol, LDL-c; low-density-lipoprotein cholesterol, FAC; functional aerobic capacity, Mets; metabolic syndrome.

ESM Table 11. Characteristics of studies included in systematic review of muscular strength

Study	Country (study name)	Numbers analysed, description and recruitment period of cohort	Men (%) Ethnicity (%)	Age at baseline (years)	Follow-up (years)	Outcome assessment	Cumulative type 2 diabetes incidence	Muscular strength assessment	Estimates from manuscript used in meta-analysis (RR/OR/HR with 95% CI)	Model control
Katzmarzyk et al., 2007 [3]	Canada<br><br>Canadian Physical Activity Longitudinal Study	865<br><br>Participants in the Canada Fitness Survey and/or Campbell's Survey of Well-being in Canada. Sampled to be representative of the Canadian population<br><br>1988	46 %<br><br>Caucasian	Mean (SD): 37.1 (12.2)<br><br>Range: 18 – 69	Mean: 15.5	Self-report	5.0 %<br><br>43 / 865<br><br>(calculated based on assumption of identical incidence in sample with data)	Maximal HGS<br><br>Dynamometer	OR per SD increase (kg)<br><br>Multivariable - BMI 0.62 (0.33 – 1.20)	Age, sex, smoking, alcohol intake, parental history of diabetes
Wander et al., 2011 [25]	USA<br><br>Japanese-American Community Diabetes Study	394<br><br>Second- and third-generation Japanese Americans of 100% Japanese ancestry<br><br>Unclear	53 %<br><br>Asian	Mean: 51.9<br><br>Range: 34-75	Range: 10-11	Clinical assessment	18.5 %<br><br>73 / 394	Maximal HGS<br><br>Dynamometer	OR per 10-pound increase<br><br>Multivariable + BMI 1.00 (0.96 – 1.04)	Age, family history of diabetes, sex, BMI
Leong et al., 2015 [26]	International<br><br>Prospective Urban-Rural	139,691<br><br>Representative samples of communities from 17	42 %<br><br>Participants from North America,	Median (IQR): 50 (42-58)	Median (IQR): 4 (2.9 – 5.1)	Records linkage and self-report	2.1 %<br><br>2939 / 139,691	Maximal HGS<br><br>Dynamometer	HR per 5-kg decrease<br><br>Multivariable + BMI 1.04 (1.01 – 1.08)	Age, sex, education level, employment status, physical activity, tobacco use, alcohol use, energy intake, % energy from

	Epidemiology Study (PURE)	countries of low to high income<br><br>2003-2009	South America, Europe, Africa, Asia							protein, community, waist-hip ratio, BMI
Crump et al., 2016 [12]	Sweden<br><br>Swedish Military Conscription Registry Study	1,534,425<br><br>Men participating in military conscription examination (97-98% of Swedish men)<br><br>1969 - 1997	100%<br><br>Caucasian	All 18	Mean: 25.7<br><br>Up to 40	Records linkage	2.2 %<br><br>34,008 / 1,534,425	Weighted composite of maximal HGS, knee extension and elbow flexion<br><br>Dynamometer	HR per 1 N/kg increase<br><br>Multivariable + BMI<br>0.97 (0.96 – 0.97)	Year of military conscription examination, CRF, family history of diabetes, education, neighbourhood socioeconomic status, BMI
Cuthbertson et al., 2016 [28]	UK<br><br>English Longitudinal study of Ageing	5953<br><br>Nationally representative sample og the English population born on or before 1952<br><br>2004/2005	45 %<br><br>Caucasian	Mean: 66 (9.4)	Median: 5.9<br><br>Range: 2- 6	Self-report	3.6%<br><br>216 / 5953	Maximal HGS<br><br>Dynamometer	HR per SD increase (kg/ kg body-weight) (additional estimates provided following personal communication)<br><br>Multivariate-adjusted - BMI<br>0.59 (0.50 – 0.69)<br><br>Multivariate-adjusted + BMI<br>0.78 (0.64 – 0.95)	Age, sex, physical activity, smoking, alcohol, depressive symptoms, prevalent CVD.
Larsen et al., 2016 [29]	USA<br><br>The Health, Aging, and Body Composition Study	2166<br><br>Random sample of Caucasian Medicare beneficiaries and all age-eligible black community residents in selected Pittsburgh and	47%<br><br>61 % Caucasian<br><br>39% Black	Approx Mean (SD): 73.8 (2.9)<br><br>Range: 70-79	Median: 11.3<br><br>Up to 14	Clinical assessment + self-report	12.2%<br><br>265 / 2166	Maximal HGS<br><br>Dynamometer	HR per SD increase (kg)<br><br>Women:<br>Multivariable - BMI<br>1.17 (0.99 – 1.38)<br><br>Multivariable + BMI<br>1.12 (0.94 – 1.33)<br><br>Men:<br>Multivariable - BMI<br>0.89 (0.75 – 1.07)	Age, ethnicity, clinical site, physical activity, smoking, lipids, hypertension, visceral fat (DXA), total body fat (DXA), BMI

		Memphis communities<br><br>1997- 1998							Multivariable + BMI<br>0.90 (0.74 –1.08)	
Li et al., 2016 [27]	Australia<br><br>Men Androgen Inflammation Lifestyle Environment and Stress (MAILES)	1632<br><br>Population-based random samples from the Florey Adelaide Male Ageing Study (FAMAS) and the North West Adelaide Health Study (NWAHS)<br><br>2002-2006	100 %<br><br>Caucasian	Mean (SD): 54.1 (11.4)	Median (IQR): 4.95 (4.4 – 5.0)	Clinical assessment	8.9 %<br><br>146 / 1632	Maximal HGS<br><br>Dynamometer	HR relative to least fit quartile of kg / kg body-weight (additional estimates provided following personal communication)<br><br>Multivariable-- BMI<br>1<br>0.58 (0.37 – 0.90)<br>0.45 (0.27 – 0.73)<br>0.28 (0.15 – 0.50)<br><br>Multivariable + BMI<br>1<br>0.70 (0.43 – 1.12)<br>0.61 (0.35 – 1.04)<br>0.44 (0.21 – 0.87)	Age, sub-cohort, income, physical activity, family history of diabetes, hypertension, BMI
Marquez-Vidal et al., 2017 [30]	Switzerland<br><br>Cohorte Lausannoise (CoLaus)	2318<br><br>Random sample from the city of Lausanne. Only individuals above the age of 50 considered for muscular strength assessment<br><br>2003	42 %<br><br>Caucasian	Mean (SD): 60.2 (6.7	1st follow-up: 5.5 years (n=2318)<br><br>2nd follow-up: 10.7 years (n=1802)	Clinical assessment	13.4 %<br><br>321 / 2318	Maximal HGS<br><br>Dynamometer	HR per 5-kg increase<br><br>Multivariable + BMI<br>0.87 (0.78 – 0.97)	Age, sex**,** BMI
Karvonen-Gutierrez et al., 2018 [31]	USA<br><br>Study of Women's Health Across the	424<br><br>Women in Michigan with intact uterus, no use of exogenous	0%<br><br>40% Caucasian<br><br>60% Black	Mean (SD): 46.4 (2.8)	Median: 8.7	Clinical assessment	37.0 %<br><br>157 / 424	Maximal HGS<br><br>Dynamometer	HR per 0.1 kg/kg body-weight increase<br><br>Multivariable – waist/hip ratio<br>0.75 (0.65 – 0.86)	Age, race/ethnicity, difficulty paying for basics, smoking status, menopausal status, exogenous hormone use, physical activity (waist/hip ratio

	Nation (SWAN)	hormones and at least one menstrual period in last 3 months. Black women were oversampled<br><br>1996							Multivariable + waist/hip ratio<br>0.81 (0.70 – 0.94)	model only), waist/hip ratio
Lee et al., 2018 (abstract only) [32]	USA<br><br>Aerobics Center longitudinal Study (ACLS)	4681<br><br>1980 - 2006	Unclear<br><br>ACLS is predominantly white males	Mean (SD): 43.3 (9.5)<br><br>Range: 18-100	Median (range): 6 (1.0 – 24.9)	Clinical assessment	4.9 %<br><br>229 / 4681	Combined 1-RM leg and bench press	HR per SD increase (kg/ kg body-weight) (additional estimates provided following personal communication)<br><br>Multivariate-adjusted - BMI<br>1.07 (0.94 – 1.22)<br><br>Multivariate-adjusted + BMI<br>1.07 (0.94 – 1.22)	Age, sex, smoking, alcohol consumption, parental history of diabetes, hypertension, hypercholesterolemia, abnormal electrocardiogram, glucose levels, physical activity, CRF, BMI
Momma et al., 2018 [33]	Japan<br><br>Niigata Wellness Study	21,784<br><br>2001-2008<br><br>Individuals participating in annual law-required health-examinations by the Niigata Association of Occupational Health in Niigata, Japan	69 %<br><br>Asian	Mean (SD): 50 (9.0)	Median: 5	Clinical assessment	4.0 %<br><br>861 / 21,784	Maximal HGS<br><br>Dynamometer	HR per SD increase (kg/ kg body-weight) (additional estimates provided following personal communication)<br><br>Multivariate-adjusted – BMI<br>*Men*<br>0.68 (0.63 – 0.73)<br>*Women*<br>0.67 (0.54 – 0.79)<br><br>Multivariate-adjusted + BMI<br>*Men*<br>0.80 (0.73 – 0.86)<br>*Women*<br>0.81 (0.73 – 0.88)	Age, smoking, alcohol consumption, breakfast skipping, hypertension, dyslipidemia, BMI
McGrath et al., 2017 [34]	USA	1383	41 %	Approx	Up to 19 years	Self-report	Unclear	Maximal HGS	*Data not harmonizable for*	Education, Employment,

	Hispanic Established Population for the Epidemiological Study of the Elderly	(using data from sensitivity analysis)<br><br>Representative sample of non-institutionalized elderly Mexican Americans in five southern US states<br><br>1993-1994	Hispanic	Mean (SD): 73.3 (6.5)				Dynamometer	*inclusion in meta-analysis*<br>HR for T2D for ”weak” relative to “strong”<br><br>Men (weak: ≤0.46 kg/kg):<br>1.05 (1.02 – 1.09)<br><br>Women (weak: ≤0.30 kg/kg):<br>1.38 (1.35 – 1.41)	Instrumental-Activities-of-the-daily living disability, Interview language, marriage status, obesity
Zhang et al., 2018 [35]	China<br><br>National Physical Education Program, Tianjin	328<br><br>2013	48 %<br><br>Asian	Mean (SD): 68 (6.1)	Mean: 3	Clinical assessment	17.1 %<br><br>56 / 328	Maximal HGS<br><br>Dynamometer	*Data not harmonizable for inclusion in meta-analysis*<br>OR for T2D per unknown increase (kg/kg body-weight)<br><br>Unadjusted<br>0.97 (0.93 – 1.00)<br><br>Multivariable (unknown)-adjusted<br>0.88 (0.82 – 0.94)	Unclear

HGS; handgrip strength, RR; relative risk, OR; odds ratio, HR; hazard ratio, SD; standard deviation, BMI; body-mass index, IQR: inter-quartile-range

ESM Table 12. Risk difference associated with a 1-MET increase in cardiorespiratory fitness or a 1-SD increase in muscular strength in age-strata and for the total U.S. adult population

	Risk difference per 100,000 people per year	95% Confidence Interval*
18+ years		
Cardiorespiratory fitness (adiposity-controlled)	54	40 to 68
Cardiorespiratory fitness (non-adiposity controlled)	134	100 to 170
Muscular strength (adiposity-controlled)	87	27 to 129
Muscular strength (non-adiposity controlled)	161	60 to 244
18-44 years		
Cardiorespiratory fitness (adiposity-controlled)	25	18 to 34
Cardiorespiratory fitness (non-adiposity controlled)	62	42 to 84
Muscular strength (adiposity-controlled)	40	12 to 62
Muscular strength (non-adiposity controlled)	74	3 to 117
44-64 years		
Cardiorespiratory fitness (adiposity-controlled)	87	64 to 112
Cardiorespiratory fitness (non-adiposity controlled)	218	150 to 280
Muscular strength (adiposity-controlled)	142	43 to 211
Muscular strength (non-adiposity controlled)	262	98 to 399
65+ years		
Cardiorespiratory fitness (adiposity-controlled)	75	54 to 98
Cardiorespiratory fitness (non-adiposity controlled)	188	127 to 246
Muscular strength (adiposity-controlled)	122	37 to 184
Muscular strength (non-adiposity controlled)	226	8 to 348

Background incidence based in 2015 U.S. [60] *Calculated based on Excel-macro described in Newcombe et al., 2014 [61].

ESM Table 13. Omitting, in turn, one study at a time from linear dose-response meta-analysis of cardiorespiratory fitness estimates including control for adiposity

Study omitted	RR	95% Confidence interval
Sui et al., 2008 [4]	0.92	0.90 to 0.95
Sieverdes et al., 2010 [6]	0.93	0.91 to 0.95
Kuwahara et al., 2014 [8]	0.92	0.90 to 0.94
Juraschek et al., 2015 [9]	0.91	0.89 to 0.94
Zaccardi et al., 2015 [10]	0.92	0.90 to 0.94
Bantle et al., 2016 [11]	0.92	0.89 to 0.94
Crump et al., 2016 [12]	0.92	0.89 to 0.95
Holtermann et al., 2017 [13]	0.92	0.89 to 0.94
Kokkinos et al., 2017 [14]	0.92	0.90 to 0.95
Momma et al., 2017 [15]	0.92	0.90 to 0.95

ESM Table 14. Omitting, in turn, one study at a time from linear dose-response meta-analysis of cardiorespiratory fitness estimates excluding control for adiposity

Study omitted	RR	95% Confidence Interval
Katzmarzyk et al., 2007 [3]	0.81	0.76 to 0.86
Sui et al., 200 8[4]	0.81	0.76 to 0.86
Sieverdes et al., 2010 [6]	0.82	0.77 to 0.87
Carnathon et al., 2009 (Men) [5]	0.82	0.77 to 0.87
Carnathon et al., 2009 (Women) [5]	0.82	0.77 to 0.87
Skretteberg et al., 2013 [7]	0.80	0.75 to 0.86
Kuwahara et al., 2014 [8]	0.79	0.74 to 0.85
Juraschek et al., 2015 [9]	0.79	0.74 to 0.84
Holtermann et al., 2017 [13]	0.79	0.74 to 0.85
Kokkinos et al., 2017 [14]	0.80	0.75 to 0.86
Kawakami et al., 2018 [16]	0.79	0.73 to 0.86

ESM Table 15. Omitting, in turn, one study at a time from linear dose-response meta-analysis of muscular strength estimates including control for adiposity

Study omitted	RR	95% Confidence Interval
Wander et al., 2011 [25]	0.86	0.80 to 0.92
Leong et al., 2015 [26]	0.87	0.80 to 0.94
Crump et al., 2016 [12]	0.88	0.81 to 0.95
Cuthbertson et al., 2016 [28]	0.88	0.80 to 0.95
Larsen et al., 2016 (Men) [29]	0.87	0.80 to 0.94
Larsen et al., 2016 (Women) [29]	0.86	0.80 to 0.92
Li et al., 2016 [27]	0.88	0.82 to 0.95
Marques-Vidal et al., 2017 [30]	0.88	0.82 to 0.95
Karvonen-Gutierrez et al., 2018 [31]	0.88	0.81 to 0.95
Lee et al., 2018 [32]	0.86	0.79 to 0.92
Momma et al., 2018 (Men)[33]	0.88	0.81 to 0.95
Momma et al., 2018 (Women) [33]	0.88	0.81 to 0.95

ESM Table 16. Omitting, in turn, one study at a time from linear dose-response meta-analysis of muscular strength estimates excluding control for adiposity

Study omitted	RR	95% Confidence Interval
Katzmarzyk et al., 2007 [3]	0.77	0.64 to 0.92
Cuthbertson et al., 2016 [28]	0.79	0.65 to 0.94
Larsen et al., 2016 (Men) [29]	0.75	0.62 to 0.90
Larsen et al., 2016 (Women) [29]	0.72	0.61 to 0.85
Li et al., 2016 [27]	0.79	0.66 to 0.95
Karvonen-Gutierrez et al., 2018 [31]	0.77	0.63 to 0.93
Lee et al., 2018 [32]	0.73	0.62 to 0.86
Momma et al., 2018 (Men) [33]	0.77	0.62 to 0.95
Momma et al., 2018 (Women) [33]	0.77	0.64 to 0.94

ESM Figure 1. Study-specific relative risks per 1-MET increase in cardiorespiratory fitness in model not controlling for adiposity

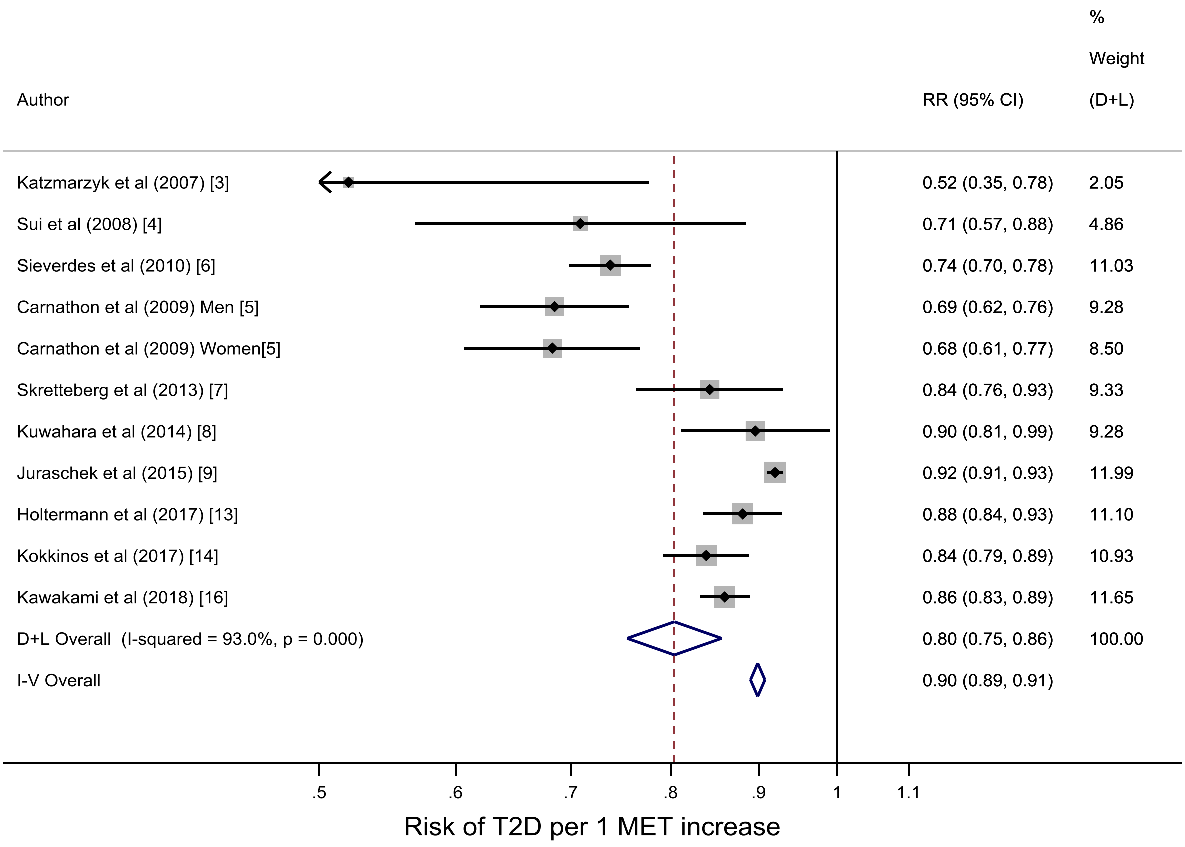

Study weights are from the random-effects analysis (D+L). Pooled RRs from the random-effects analysis (D + L) and the fixed-effects analysis (I-V) are shown based on 10 cohorts providing non-adiposity controlled estimates. Four of these cohorts provided per 1-MET (or ml O2 kg−1 min−1, converted to METs) [3, 5, 7, 9] estimates while the linear estimate was modelled using GLST in 6 studies [4, 6, 8, 13, 14, 16]. D+L; DerSimonian and Laird (random-effects model), I-V; inverse variance (fixed effects-model).

ESM Figure 2. Relative risk of type 2 diabetes with increasing cardiorespiratory fitness modelled using restricted cubic splines. Estimates are not controlled for adiposity

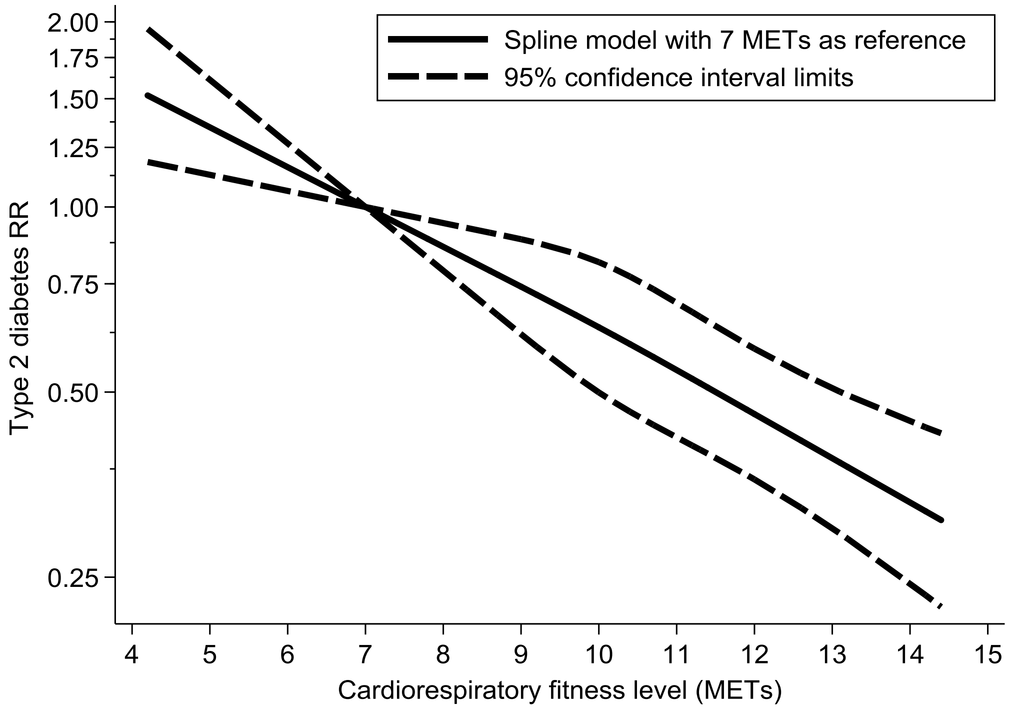

ESM Figure 3. Study-specific relative risks per standard deviation increase in muscular strength in model not controlling for adiposity

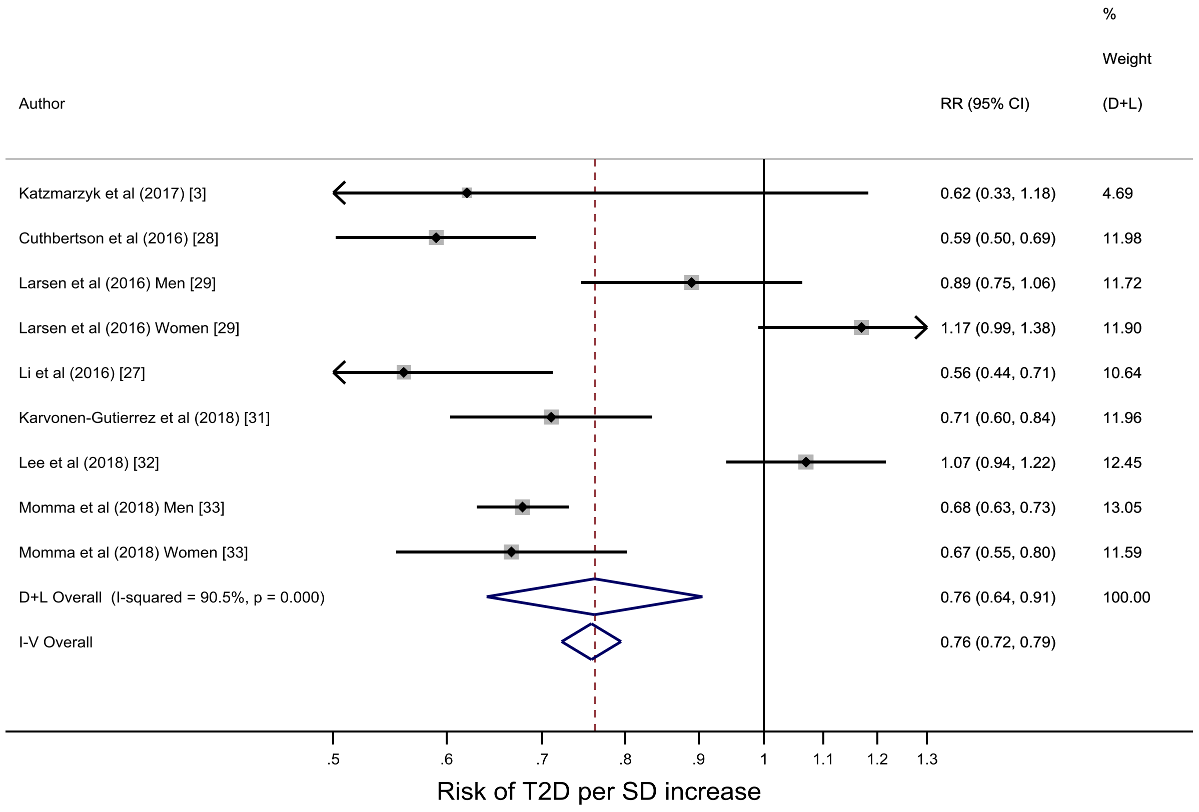

Study weights are from the random-effects analysis (D+L). Pooled RRs from the random-effects analysis (D + L) and the fixed-effects analysis (I-V) are shown based on 7 cohorts providing non-adiposity controlled estimates. Six of these cohorts provided per unit estimates (harmonized to per SD) [3, 28, 29, 31, 32, 33] while the linear estimate was modelled using GLST in 1 study [27]. D+L; DerSimonian and Laird (random-effects model), I-V; inverse variance (fixed effects-model).

ESM Figure 4. Risk of small-study bias visualized by funnel-plot of cardiorespiratory fitness estimates including control for adiposity

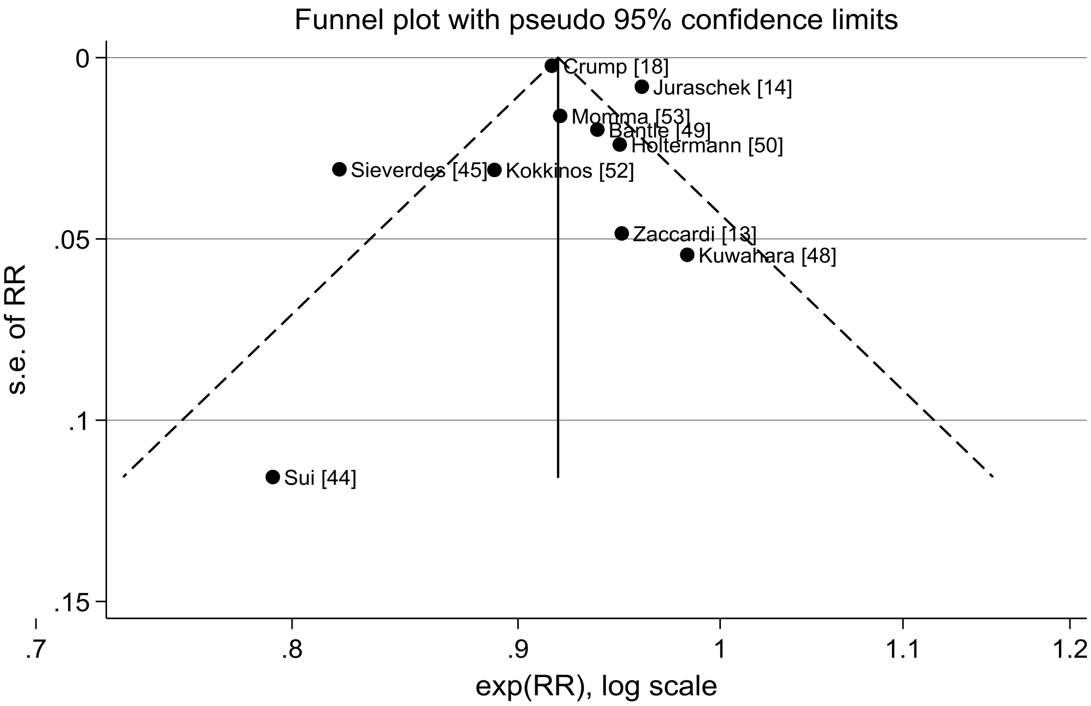

ESM Figure 5. Risk of small-study bias visualized by funnel-plot of cardiorespiratory fitness estimates excluding control for adiposity

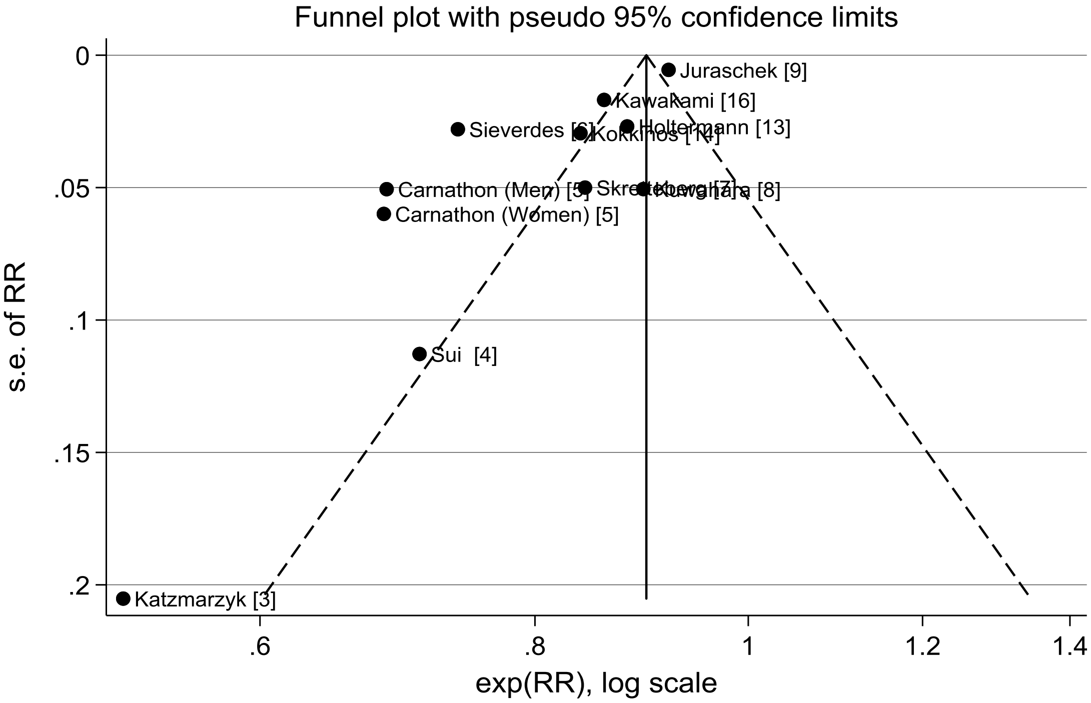

ESM Figure 6. Risk of small-study bias visualized by funnel-plot of muscular strength estimates including control for adiposity

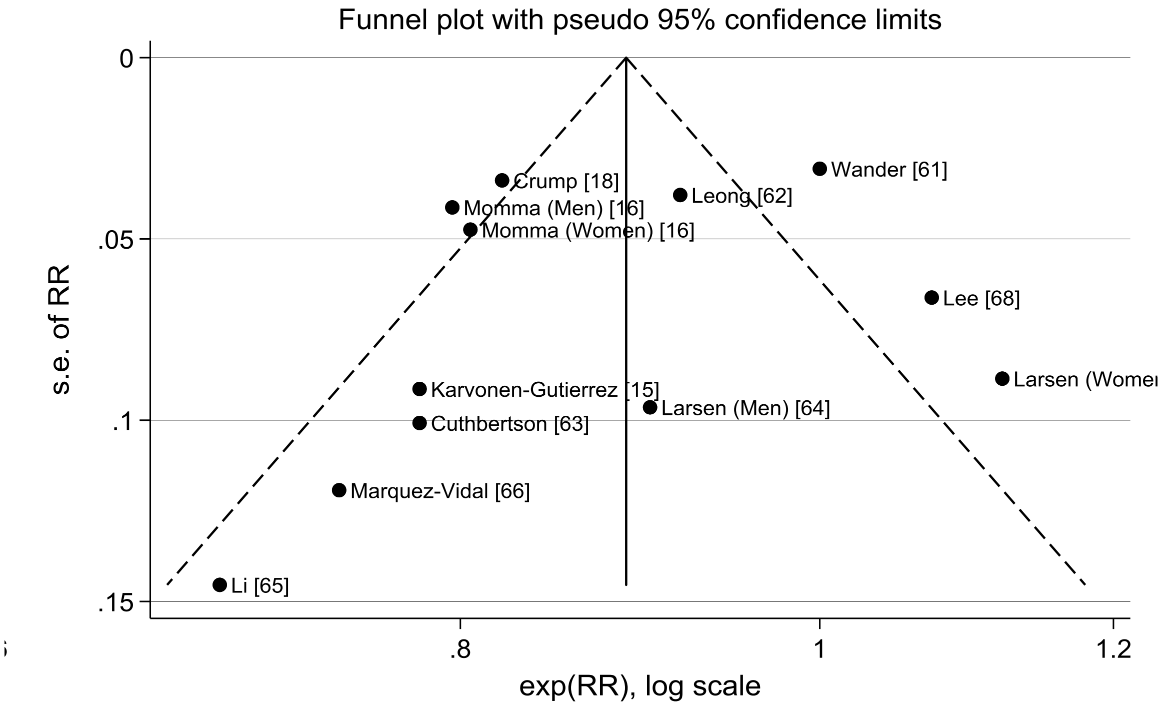

ESM Figure 7. Risk of small-study bias visualized by funnel-plot of muscular strength estimates excluding control for adiposity

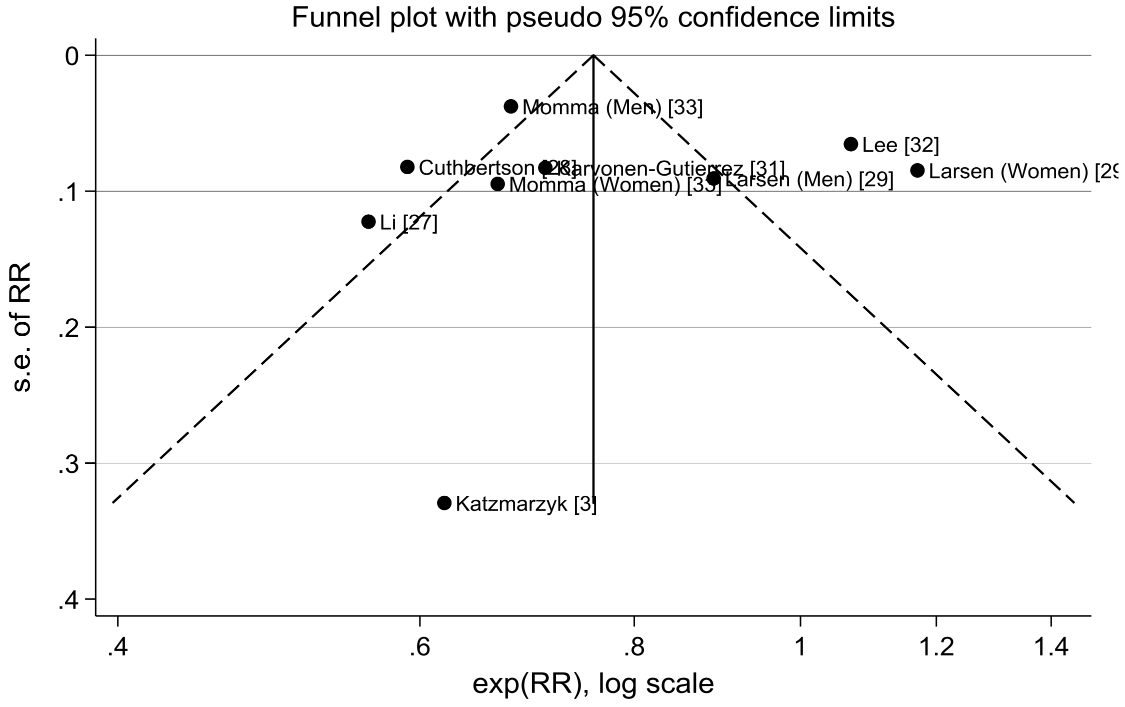

References

[1] American College of Sports Medicine (2018) ACSM's guidelines for exercise testing and prescription. 10th Edition. Wolters Kluwer Health, Philadelphia, PA

[2] Lynch J, Helmrich SP, Lakka TA, et al. (1996) Moderately intense physical activities and high levels of cardiorespiratory fitness reduce the risk of non-insulin-dependent diabetes mellitus in middle-aged men. Arch Intern Med 156: 1307-1314

[3] Katzmarzyk PT, Craig CL, Gauvin L (2007) Adiposity, physical fitness and incident diabetes: the physical activity longitudinal study. Diabetologia 50: 538-544

[4] Sui X, Hooker SP, Lee IM, et al. (2008) A prospective study of cardiorespiratory fitness and risk of type 2 diabetes in women. Diabetes care 31: 550-555

[5] Carnethon MR, Sternfeld B, Schreiner PJ, et al. (2009) Association of 20-year changes in cardiorespiratory fitness with incident type 2 diabetes: the coronary artery risk development in young adults (CARDIA) fitness study. Diabetes care 32: 1284-1288

[6] Sieverdes JC, Sui X, Lee DC, et al. (2010) Physical activity, cardiorespiratory fitness and the incidence of type 2 diabetes in a prospective study of men. Br J Sports Med 44: 238-244

[7] Skretteberg PT, Grytten AN, Gjertsen K, et al. (2013) Triglycerides-diabetes association in healthy middle-aged men: modified by physical fitness? A long term follow-up of 1962 Norwegian men in the Oslo Ischemia Study. Diabetes Res Clin Pract 101: 201-209

[8] Kuwahara K, Uehara A, Kurotani K, et al. (2014) Association of cardiorespiratory fitness and overweight with risk of type 2 diabetes in Japanese men. PLoS One 9: e98508

[9] Juraschek SP, Blaha MJ, Blumenthal RS, et al. (2015) Cardiorespiratory fitness and incident diabetes: the FIT (Henry Ford ExercIse Testing) project. Diabetes care 38: 1075-1081

[10] Zaccardi F, O'Donovan G, Webb DR, et al. (2015) Cardiorespiratory fitness and risk of type 2 diabetes mellitus: A 23-year cohort study and a meta-analysis of prospective studies. Atherosclerosis 243: 131-137

[11] Bantle AE, Chow LS, Steffen LM, et al. (2016) Association of Mediterranean diet and cardiorespiratory fitness with the development of pre-diabetes and diabetes: the Coronary Artery Risk Development in Young Adults (CARDIA) study. BMJ Open Diabetes Res Care 4: e000229

[12] Crump C, Sundquist J, Winkleby MA, Sieh W, Sundquist K (2016) Physical fitness among Swedish military conscripts and long-term risk for type 2 diabetes mellitus: a cohort study. Ann Intern Med 164: 577-584

[13] Holtermann A, Gyntelberg F, Bauman A, Thorsten Jensen M (2017) Cardiorespiratory fitness, fatness and incident diabetes. Diabetes Res Clin Pract 134: 113-120

[14] Kokkinos P, Faselis C, Narayan P, et al. (2017) Cardiorespiratory Fitness and Incidence of Type 2 Diabetes in United States Veterans on Statin Therapy. Am J Med 130: 1192-1198

[15] Momma H, Sawada SS, Lee IM, et al. (2017) Consistently High Level of Cardiorespiratory Fitness and Incidence of Type 2 Diabetes. Med Sci Sports Exerc 49: 2048-2055

[16] Kawakami R, Sawada SS, Lee IM, et al. (2018) Long-term Impact of Cardiorespiratory Fitness on Type 2 Diabetes Incidence: A Cohort Study of Japanese Men. J Epidemiol 28: 266-273

[17] Ohlson LO, Larsson B, Bjorntorp P, et al. (1988) Risk factors for type 2 (non-insulin-dependent) diabetes mellitus. Thirteen and one-half years of follow-up of the participants in a study of Swedish men born in 1913. Diabetologia 31: 798-805

[18] Williams PT (2008) Relationship of running intensity to hypertension, hypercholesterolemia, and diabetes. Med Sci Sports Exerc 40: 1740-1748

[19] Kinney GL, Baker E, Klein OL, et al. (2014) Incident diabetes mellitus in heavy smokers. Diabetes 1): A365

[20] Someya Y, Kawai S, Kohmura Y, Aoki K, Daida H (2014) Cardiorespiratory fitness and the incidence of type 2 diabetes: a cohort study of Japanese male athletes. BMC Public Health 14: 493
[21] Jae SY, Franklin BA, Choo J, Yoon ES, Choi YH, Park WH (2016) Fitness, Body Habitus, and the Risk of Incident Type 2 Diabetes Mellitus in Korean Men. Am J Cardiol 117: 585-589
[22] Sydo N, Sydo T, Carta KG, et al. (2016) Cardiovascular fitness reduces the risk of weight-associated comorbidities and all-cause mortality in past smokers. Journal of the American College of Cardiology 1): 1858
[23] Wu CJ, Kao TW, Yang HF, et al. (2018) Predictability of cardiorespiratory fitness on the risk of developing metabolic syndrome and diabetes mellitus in Taiwan adults: preliminary analysis of a cohort study. Obes Res Clin Pract 12: 541–546
[24] Wells GA, Shea B, O'Connell D, et al. (2009) The Newcastle-Ottawa Scale (NOS) for assessing the quality in nonrandomized studies in meta-analyses. Available from http://www.ohri.ca/programs/clinical_epidemiology/oxford.asp, accessed February 2018
[25] Wander PL, Boyko EJ, Leonetti DL, McNeely MJ, Kahn SE, Fujimoto WY (2011) Greater hand-grip strength predicts a lower risk of developing type 2 diabetes over 10 years in leaner Japanese Americans. Diabetes Res Clin Pract 92: 261-264
[26] Leong DP, Teo KK, Rangarajan S, et al. (2015) Prognostic value of grip strength: findings from the Prospective Urban Rural Epidemiology (PURE) study. Lancet 386: 266-273
[27] Li JJ, Wittert GA, Vincent A, et al. (2016) Muscle grip strength predicts incident type 2 diabetes: Population-based cohort study. Metabolism 65: 883-892
[28] Cuthbertson DJ, Bell JA, Ng SY, Kemp GJ, Kivimaki M, Hamer M (2016) Dynapenic obesity and the risk of incident Type 2 diabetes: the English Longitudinal Study of Ageing. Diabet Med 33: 1052-1059
[29] Larsen BA, Wassel CL, Kritchevsky SB, et al. (2016) Association of Muscle Mass, Area, and Strength With Incident Diabetes in Older Adults: The Health ABC Study. J Clin Endocrinol Metab 101: 1847-1855
[30] Marques-Vidal P, Vollenweider P, Waeber G, Jornayvaz FR (2017) Grip strength is not associated with incident type 2 diabetes mellitus in healthy adults: The CoLaus study. Diabetes Res Clin Pract 132: 144-148
[31] Karvonen-Gutierrez CA, Peng Q, Peterson M, Duchowny K, Nan B, Harlow S (2018) Low grip strength predicts incident diabetes among mid-life women: the Michigan Study of Women's Health Across the Nation. Age Ageing 47: 685–691
[32] Lee DC, Brellenthin A, Sui X, Blair S (2018) Muscular strength and type 2 diabetes prevention. Circulation 137: MP32 (Abstract)
[33] Momma H, Sawada SS, Kato K, et al. (2018) Physical Fitness Tests and Type 2 Diabetes Among Japanese: A Longitudinal Study From the Niigata Wellness Study. J Epidemiol
[34] McGrath RP, Vincent BM, Snih SA, et al. (2017) The Association Between Handgrip Strength and Diabetes on Activities of Daily Living Disability in Older Mexican Americans. J Aging Health 30: 1305–1318
[35] Zhang W, Yang X, Han P, et al. (2018) Risk Factors for Developing Diabetes among Community Dwelling Elderly with Impaired Fasting Glycaemia after 3 Years. J Diabetes
[36] Carnethon MR, Gidding SS, Nehgme R, Sidney S, Jacobs DR, Jr., Liu K (2003) Cardiorespiratory fitness in young adulthood and the development of cardiovascular disease risk factors. JAMA 290: 3092-3100
[37] Wei M, Gibbons LW, Mitchell TL, Kampert JB, Lee CD, Blair SN (1999) The association between cardiorespiratory fitness and impaired fasting glucose and type 2 diabetes mellitus in men. Ann Intern Med 130: 89-96
[38] Le TD, Bae S, Ed Hsu C, Singh KP, Blair SN, Shang N (2008) Effects of Cardiorespiratory Fitness on Serum Ferritin Concentration and Incidence of Type 2 Diabetes: Evidence from the Aerobics Center Longitudinal Study (ACLS). Rev Diabet Stud 5: 245-252
[39] Lee DC, Sui X, Church TS, Lee IM, Blair SN (2009) Associations of cardiorespiratory fitness and obesity with risks of impaired fasting glucose and type 2 diabetes in men. Diabetes care 32: 257-262

[40] Goodrich KM, Crowley SK, Lee DC, Sui XS, Hooker SP, Blair SN (2012) Associations of cardiorespiratory fitness and parental history of diabetes with risk of type 2 diabetes. Diabetes Res Clin Pract 95: 425-431
[41] Radford NB, DeFina LF, Barlow CE, et al. (2015) Effect of fitness on incident diabetes from statin use in primary prevention. Atherosclerosis 239: 43-49
[42] Sloan RA, Haaland BA, Sawada SS, et al. (2016) A Fit-Fat Index for Predicting Incident Diabetes in Apparently Healthy Men: A Prospective Cohort Study. PLoS One 11: e0157703
[43] Sawada SS, Lee IM, Muto T, Matuszaki K, Blair SN (2003) Cardiorespiratory fitness and the incidence of type 2 diabetes: prospective study of Japanese men. Diabetes care 26: 2918-2922
[44] Sawada SS, Lee IM, Naito H, et al. (2010) Long-term trends in cardiorespiratory fitness and the incidence of type 2 diabetes. Diabetes care 33: 1353-1357
[45] Sawada SS, Lee IM, Naito H, Tsukamoto K, Muto T, Blair SN (2010) Muscular and performance fitness and the incidence of type 2 diabetes: prospective study of Japanese men. J Phys Act Health 7: 627-632
[46] Kawakami R, Sawada SS, Matsushita M, et al. (2014) Reference values for cardiorespiratory fitness and incidence of type 2 diabetes. J Epidemiol 24: 25-30
[47] Sloan RA, Sawada SS, L IM, et al. (2018) The Association of Fit-Fat Index with Incident Diabetes in Japanese Men: A Prospective Cohort Study. Sci Rep 8: 569
[48] Narayan P, Faselis CC, Pittaras A, et al. (2016) Statin therapy, fitness status and risk of type 2 diabetes in hypertensive patients. Journal of the American Society of Hypertension 10 (4 Supplement): e32
[49] Bjornholt JV, Erikssen G, Liestol K, Jervell J, Erikssen J, Thaulow E (2001) Prediction of Type 2 diabetes in healthy middle-aged men with special emphasis on glucose homeostasis. Results from 22.5 years' follow-up. Diabet Med 18: 261-267
[50] Chene G, Thompson SG (1996) Methods for summarizing the risk associations of quantitative variables in epidemiologic studies in a consistent form. Am J Epidemiol 144: 610-621
[51] Fletcher GF, Balady G, Froelicher VF, Hartley LH, Haskell WL, Pollock ML (1995) Exercise standards. A statement for healthcare professionals from the American Heart Association. Writing Group. Circulation 91: 580-615
[52] Sidney S, Haskell WL, Crow R, et al. (1992) Symptom-limited graded treadmill exercise testing in young adults in the CARDIA study. Med Sci Sports Exerc 24: 177-183
[53] Grontved A, Ried-Larsen M, Ekelund U, Froberg K, Brage S, Andersen LB (2013) Independent and combined association of muscle strength and cardiorespiratory fitness in youth with insulin resistance and beta-cell function in young adulthood: the European Youth Heart Study. Diabetes care 36: 2575-2581
[54] Hamling J, Lee P, Weitkunat R, Ambuhl M (2008) Facilitating meta-analyses by deriving relative effect and precision estimates for alternative comparisons from a set of estimates presented by exposure level or disease category. Stat Med 27: 954-970
[55] The Cochrane Collaboration. Higgins J & Green S (Editors) (2011) Cochrane Handbook for Systematic Reviews of Interventions. Table 7.7.a: Formulae for combining groups. Available from http://handbook-5-1.cochrane.org/, accessed November 2017
[56] Kaminsky LA, Arena R, Myers J (2015) Reference Standards for Cardiorespiratory Fitness Measured With Cardiopulmonary Exercise Testing: Data From the Fitness Registry and the Importance of Exercise National Database. Mayo Clin Proc 90: 1515-1523
[57] Aspenes ST, Nilsen TI, Skaug EA, et al. (2011) Peak oxygen uptake and cardiovascular risk factors in 4631 healthy women and men. Med Sci Sports Exerc 43: 1465-1473
[58] Lee IM, Shiroma EJ, Lobelo F, Puska P, Blair SN, Katzmarzyk PT (2012) Effect of physical inactivity on major non-communicable diseases worldwide: an analysis of burden of disease and life expectancy. Lancet 380: 219-229
[59] Rehm J, Taylor B, Patra J, Gmel G (2006) Avoidable burden of disease: conceptual and methodological issues in substance abuse epidemiology. Int J Methods Psychiatr Res 15: 181-191

[60] Centers for Disease Control and Prevention (CDC) (2017) National Diabetes Statistics Report, 2017: Estimates of Diabetes and Its Burden in the United States. Available from https://www.cdc.gov/diabetes/pdfs/data/statistics/national-diabetes-statistics-report.pdf, accessed October 2017
[61] Newcombe RG, Bender R (2014) Implementing GRADE: calculating the risk difference from the baseline risk and the relative risk. Evid Based Med 19: 6-8
